# Identification of recurrent *FHL2-GLI2* oncogenic fusion in sclerosing stromal tumors of the ovary

**DOI:** 10.1038/s41467-019-13806-x

**Published:** 2020-01-02

**Authors:** Sarah H. Kim, Arnaud Da Cruz Paula, Thais Basili, Higinio Dopeso, Rui Bi, Fresia Pareja, Edaise M. da Silva, Rodrigo Gularte-Mérida, Zhen Sun, Sho Fujisawa, Caitlin G. Smith, Lorenzo Ferrando, Ana Paula Martins Sebastião, Yonina Bykov, Anqi Li, Catarina Silveira, Charles W. Ashley, Anthe Stylianou, Pier Selenica, Wesley R. Samore, Achim A. Jungbluth, Dmitriy Zamarin, Nadeem R. Abu-Rustum, Kristian Helin, Robert A. Soslow, Jorge S. Reis-Filho, Esther Oliva, Britta Weigelt

**Affiliations:** 1 Department of Surgery, Memorial Sloan Kettering Cancer Center, New York, NY 10065 USA; 2 Department of Pathology, Memorial Sloan Kettering Cancer Center, New York, NY 10065 USA; 3 Department of Pathology, Fudan University Shanghai Cancer Center, 200032 Shanghai, People’s Republic of China; 4 Cell Biology Program and Center for Epigenetics, Memorial Sloan Kettering Cancer Center, New York, NY 10065 USA; 5 Molecular Cytology Core Facility, Memorial Sloan Kettering Cancer Center, New York, NY 10065 USA; 6 Department of Internal Medicine, University of Genoa, 16126 Genova, Italy; 7 Department of Medical Pathology, Federal University of Paraná, Curitiba, Paraná 80060-000 Brazil; 8 Department of Medicine, Memorial Sloan Kettering Cancer Center, New York, NY 10065 USA; 9 GenoMed SA, Institute of Molecular Medicine, University of Lisbon, 1649 Lisbon, Portugal; 10 Department of Pathology, Massachusetts General Hospital, Harvard Medical School, Boston, MA 02114 USA; 11 Biotech Research and Innovation Centre (BRIC), University of Copenhagen, 2200 Copenhagen, Denmark; 12 The Novo Nordisk Foundation for Stem Cell Biology (Danstem), University of Copenhagen, 2200 Copenhagen, Denmark

**Keywords:** Ovarian cancer, Cell signalling, DNA sequencing, Next-generation sequencing, RNA sequencing

## Abstract

Sclerosing stromal tumor (SST) of the ovary is a rare type of sex cord-stromal tumor (SCST), whose genetic underpinning is currently unknown. Here, using whole-exome, targeted capture and RNA-sequencing, we report recurrent *FHL2-GLI2* fusion genes in 65% (17/26) of SSTs and other *GLI2* rearrangements in additional 15% (4/26) SSTs, none of which are detected in other types of SCSTs (*n* = 48) or common cancer types (*n* = 9,950). The *FHL2-GLI2* fusions result in transcriptomic activation of the Sonic Hedgehog (SHH) pathway in SSTs. Expression of the FHL2-GLI2 fusion in vitro leads to the acquisition of phenotypic characteristics of SSTs, increased proliferation, migration and colony formation, and SHH pathway activation. Targeted inhibition of the SHH pathway results in reversal of these oncogenic properties, indicating its role in the pathogenesis of SSTs. Our results demonstrate that the *FHL2-GLI2* fusion is likely the oncogenic driver of SSTs, defining a genotypic–phenotypic correlation in ovarian neoplasms.

## Abstract (web summary)

Little is known about the genetics of sclerosing stromal tumor of the ovary, a rare type of sex cord-stromal tumor. Here, the authors use sequencing strategies to show that in a cohort of 26 tumor samples 65% carry a *FHL2-GLI2* fusion gene and demonstrate in vitro that the fusion gene has oncogenic properties.

## Introduction

Sex cord-stromal tumors are a heterogeneous group of benign and malignant neoplasms derived from the primitive sex cords or stromal components of the ovary^1^. Sclerosing stromal tumors (SSTs) represent an uncommon subtype of sex cord-stromal tumor (<5%)^1–3^, occurring in young adults in the second and third decades of life^4^. Patients typically present with abdominal or pelvic pain and menstrual irregularities in the setting of a unilateral pelvic mass. SSTs are usually hormonally inactive, however androgenic changes in the setting of pregnancy or virilization have been reported^4^. Although regarded as a benign neoplasm, its clinical presentation, imaging findings and elevated CA-125 levels may mimic those of malignant ovarian neoplasms, resulting in young women undergoing radical surgical resection^2,5,6^. In addition, the panoply of histologic features SSTs display may pose diagnostic challenges and result in misdiagnoses^2,7^. Immunohistochemistry may be helpful as these tumors are positive for sex cord markers including calretinin and FOXL2^8–10^; however, understanding the genetic underpinning of SSTs may aide in the diagnosis and management of these rare ovarian neoplasms.

The genomic landscape of common types of ovarian cancer has been thoroughly investigated^11^, and the genetic underpinnings of some rare types of ovarian cancer have been identified. For instance, *SMARCA4* loss-of-function mutations have been documented in small cell carcinomas of the ovary hypercalcemic type^12^ and *FOXL2* p.C134W hotspot mutations have been described in >97% of adult-type granulosa cell tumors^1,9^, the most common sex cord-stromal tumor. These seminal studies indicate the vast potential for the discovery of unique genomic drivers in rare types of ovarian tumors^13^. In addition, *DICER1* mutations have been detected in a subset of Sertoli-Leydig cell tumors and other non-epithelial ovarian cancers^14,15^. The genetic landscape of other sex cord-stromal tumors, including SSTs, however, is currently unknown.

We posited that if SSTs are driven by a pathognomonic genetic alteration, this information could be used for the development of ancillary markers to mitigate the diagnostic challenges posed by these rare tumors. In this study, we sought to define the repertoire of genetic alterations in SSTs, using a combination of whole-exome sequencing, targeted massively parallel sequencing and RNA-sequencing. Our analyses reveal the presence of a highly recurrent *FHL2-GLI2* fusion transcript or *GLI2* rearrangements in SSTs. Functional analyses in vitro establish that expression of the FHL2-GLI2 fusion increases signaling via the Sonic Hedgehog (SHH) pathway and results in the acquisition of oncogenic properties, which can be reversed through its chemical inhibition, thereby establishing a genotypic-phenotypic correlation and the importance of the SHH pathway in the biology of these tumors.

## Results

### Clinical and histologic features of SSTs

**Fig. 1:**
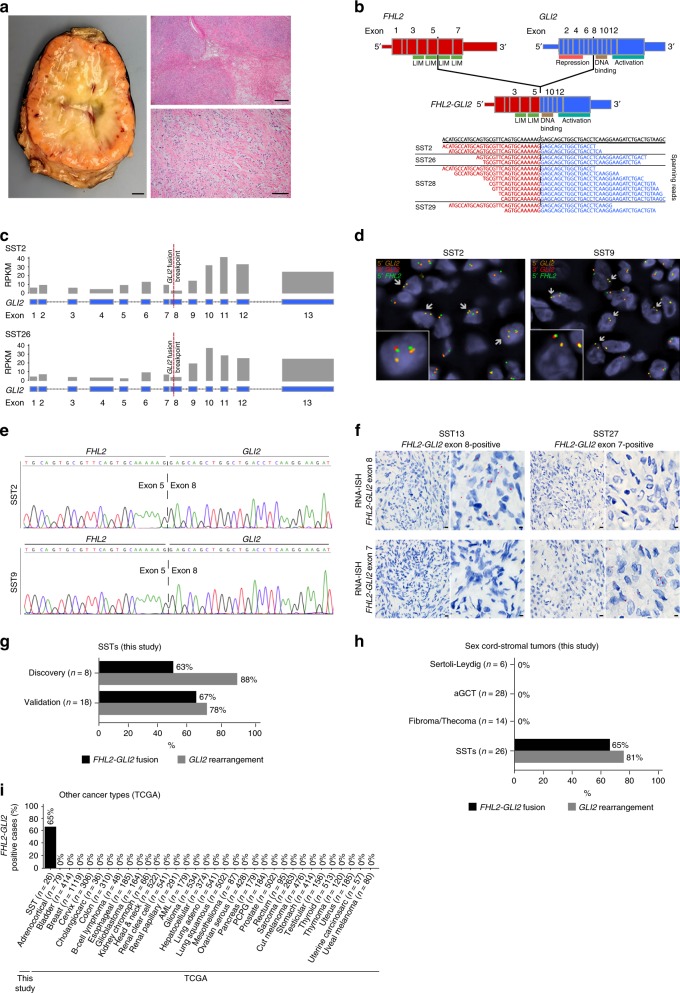
Recurrent *FHL2-GLI2* fusion gene in sclerosing stromal tumors of the ovary. **a** Photograph of the cut section of an ovarian sclerosing stromal tumor (SST; left) displaying classic SST appearance with yellow tissue at periphery and white, central fibrotic depression, and micrographs of hematoxylin & eosin stained representative section at low (top right) and high (bottom right) magnification. Scale bars, 1 cm (left), 200 μm (top right), 50 μm (bottom right). **b** Schematic representation of the *FHL2-GLI2* fusion transcript including the exons and domains involved. The breakpoint of the 5′ and 3′ partner genes are represented as black vertical lines. Spanning reads are depicted and aligned to the predicted junction sequence. **c** Schematic representation showing the Reads Per Kilobase per Million (RPKM) mapped read counts of each *GLI2* exon. The *GLI2* fusion breakpoint is represented as a red dashed line. **d** Fluorescence in situ hybridization (FISH) of two representative SSTs using a three-color *FHL2-GLI2* probe, with 5′ *GLI2* (orange), 3′ *GLI2* (red), and 5′ *FHL2* (green), showing the presence of the *FHL2-GLI2* fusion (white arrows). **e** Representative Sanger sequencing electropherograms of the genomic *FHL2-GLI2* breakpoint. **f** RNA in situ hybridization (RNA-ISH) using custom *FHL2-GLI2* probes (red) showing the chimeric *FHL2-GLI2* mRNA expression in two representative SSTs harboring the *FHL2-GLI2* fusion. **g** Frequency of the *FHL2-GLI2* fusion gene and *GLI2* rearrangements in 26 SSTs from this study. **h** Frequency of the *FHL2-GLI2* fusion gene and *GLI2* rearrangements in 26 SSTs and frequency of the *FHL2-GLI2* fusion gene in 48 other ovarian sex cord-stromal tumors from this study. aGCT, adult-type granulosa cell tumor. **i** Frequency of *FHL2-GLI2* fusion gene in 26 SSTs from this study and in 9950 tumors from 33 cancer types from The Cancer Genome Atlas (TCGA). AML acute myeloid leukemia, PCPG pheochromocytoma and paraganglioma.

SSTs were retrieved from the authors’ institutions, following approval by the institutional review boards (IRBs)/local ethics committees, and patient consents were obtained where appropriate. Following central pathology review, 26 tumors were classified as SSTs and included in this study (Supplementary Table 1, Supplementary Fig. 1). Patient median age at diagnosis was 29 (range 14–56) years, and all patients underwent surgical resection without any further adjuvant treatment (Supplementary Table 1). Histologically, SSTs were characterized by alternating areas of hypercellularity and hypocellularity imparting a vague lobulated architecture. An often prominent component of staghorn vessels, as well as varying numbers of spindle and luteinized stromal cells with overall bland cytologic features and overall low mitotic and proliferation rates were noted (Fig. 1a, Supplementary Table 1, Supplementary Fig. 2).

### SSTs display few mutations and copy number alterations

To determine whether SSTs are underpinned by a pathognomonic somatic mutation, DNA samples from eight SSTs and matched normal tissues were subjected to whole-exome sequencing (WES; *n* = 3, median depth of coverage of tumor 108×, range 50×−226×, and normal samples 22×, range 20×−144×) or to targeted massively parallel sequencing due to the limited yields of DNA available (*n* = 5, median depth of coverage of tumor samples 698×, range 423×−855×) using the Memorial Sloan Kettering-Integrated Mutation Profiling of Actionable Cancer Targets (MSK-IMPACT^16^) assay (Supplementary Fig. 1, Supplementary Table 1). We reasoned that if SSTs were to harbor a somatic mutation at the same frequency of *FOXL2* mutations in adult-type granulosa cell tumors^9^, eight samples would confer >90% of statistical power to detect a pathognomonic mutation.

SSTs displayed a low mutation burden, with a median of 16 (range 4–36) somatic mutations identified by WES, of which 9 (range 3–10) were non-synonymous. MSK-IMPACT sequencing detected a median of 2 (range 1–3) somatic mutations, with only 1 (range 0–3) being non-synonymous (Supplementary Table 2). None of the mutations identified were recurrent in the SSTs analyzed. In fact, the majority of mutations were non-pathogenic missense mutations not affecting hotspot residues, and three of the five SSTs subjected to MSK-IMPACT sequencing did not harbor any mutations affecting the 410 cancer-related genes tested (Supplementary Fig. 3). One potentially pathogenic non-synonymous somatic mutation was identified, a subclonal *ATR* (p.R1183*) nonsense mutation in SST2 (Supplementary Fig. 3, Supplementary Table 2). Genome-wide copy number analysis revealed a paucity of copy number alterations, and the eight SSTs analyzed displayed diploid/near-diploid genomes. No homozygous deletions or amplifications were detected in any of the SSTs studied (Supplementary Fig. 3).

Taken together, SSTs are characterized by a low mutation burden, low levels of genomic instability, and the lack of recurrent somatic mutations or copy number alterations.

### SSTs of the ovary harbor a recurrent *FHL2-GLI2* fusion gene

RNA-sequencing of eight SSTs revealed a recurrent in-frame *FHL2-GLI2* fusion gene with a high driver probability as defined by OncoFuse^17^ in 50% (4/8) of the cases analyzed (Supplementary Table 3). This fusion gene resulted in a chimeric transcript composed of exons 1–5 of *FHL2* (chr2:105984027) and exons 8–13 of *GLI2* (chr2:121732551), likely through a paracentric inversion of chromosome 2q (Fig. 1b). *FHL2* (Four And A Half LIM Domains 2) is a member of the four and a half LIM domain‐only family, which is constitutively expressed in ovarian tissues^18,19^, whereas the oncogene *GLI2* (GLI Family Zinc Finger 2) encodes a zinc finger transcription factor, which comprises a DNA binding domain, a GLI2 repression domain at the N-terminus and an activation domain at its C-terminus^20^ (Fig. 1b). In this fusion, two of the four LIM protein domains of *FHL2* were retained, while the repression domain of *GLI2* was lost (Fig. 1b). The fusion of *FHL2* to the 3′-end of *GLI2* resulted in increased expression of exons 9–13 of *GLI2*, including the DNA binding and activation domains (Fig. 1c). Based on the domains involved in the chimeric transcript and the patterns of exonic transcription in SSTs harboring the *FHL2-GLI2* chimeric gene (Fig. 1c), the most parsimonious explanation is that the 5′ aspects of FHL2 are juxtaposed with the DNA binding and activation domains of GLI2, coupled with a loss of the GLI2 repression domain^18,19,21^. As important mediators of the SHH pathway^22^, the GLI family proteins, GLI1–3, play an integral role in embryogenesis and are regarded as potent oncogenes^23,24^. Specifically, *GLI2* functions as a transcription factor binding DNA through zinc finger motifs^22,24^, and *GLI1* has been found to constitute an oncogenic partner in fusion genes described in gastroblastoma^25^, soft tissue sarcomas^26^, and fibromyxomas^27^.

One of the initial eight SSTs of the discovery cohort, SST27, harbored a *FHL2-GLI2* fusion involving exons 7–13 of *GLI2*, rather than exons 8–13 of *GLI2* found in the remaining 16 SSTs; this chimeric *FHL2-GLI2* transcript in SST27 also leads to a juxtaposition of the 5′ elements of *FHL2* with *GLI2* lacking the repression domain whilst leaving the DNA binding and activation domains intact (Supplementary Fig. 4a, Supplementary Table 3). In addition, SST12, which lacked the *FHL2-GLI2* fusion as assessed by RNA-sequencing and RT-PCR, was found to harbor a *DYNLL1-GLI2* fusion gene with a high driver probability as defined by OncoFuse^17^ (Supplementary Table 3), where the chimeric transcript was composed of exons 1–2 of *DYNLL1* and exons 8–13 of *GLI2* (Supplementary Fig. 4b).

To validate the presence of the *GLI2* fusion genes found by RNA-sequencing, we performed reverse transcription (RT)-PCR (for *FHL2-GLI2* exons 7–13 *GLI2*, exons 8–13 *GLI2*, and *DYNLL1-GLI2*) and/or fluorescence in situ hybridization (FISH) analysis with custom *GLI2* break-apart probes in the initial eight SSTs and an additional 18 SSTs (validation cohort), for which RNA of sufficient quantity/quality for RNA-sequencing could not be obtained. The presence of the *FHL2-GLI2* fusion gene was validated in the index cases, and 12 of the additional 18 SSTs (67%) were found to harbor an *FHL2-GLI2* fusion transcript (Figs. 1d–f, Supplementary Table 4). The only recurrent *GLI2* fusion gene identified in the 26 cases tested was the *FHL2-GLI2* comprising exons 8–13 of *GLI2* (Fig. 1a, b); the *FHL2-GLI2* containing exons 7–13 *GLI2* and *DYNLL1-GLI2* fusions were found to be restricted to SST27 and SST12, respectively (Supplementary Fig. 4). FISH analysis also revealed the presence of break-apart signals of the 5’*GLI2*–3’*GLI2* probe in 3 SSTs lacking *FHL2-GLI2* and *DYNLL1-GLI2* fusions by RNA-sequencing and/or RT-PCR (SST1, SST8, SST20), indicative of a *GLI2* rearrangement (Supplementary Fig. 5, Supplementary Table 4).

In total, 21/26 (81%) of the SSTs analyzed harbored *GLI2* rearrangements, with 16/26 (62%) harboring the *FHL2-GLI2* comprising exons 8–13 of *GLI2*, 1/26 (4%) having a *FHL2-GLI2* containing exons 7–13 *GLI2*, 1/26 (4%) displaying a *DYNLL1-GLI2* fusion gene and 3/26 (12%) harboring other forms of *GLI2* rearrangements whose partners have yet to be identified (Fig. 1g). Of note, no differences in the histologic or clinical features of the SSTs according to the presence/absence of the *GLI2* rearrangements, the type of *GLI2* rearrangements or the partner of the *GLI2* rearrangements were observed (Supplementary Table 1). Reanalysis of the whole-exome and targeted capture sequencing data of the *GLI2* wild-type SSTs failed to reveal any likely driver genetic alteration.

### *FHL2-GLI2* fusion gene as a defining feature of ovarian SSTs

To determine whether the *FHL2-GLI2* fusion gene would constitute a pathognomonic genetic alteration for SSTs, we investigated the presence of the *FHL2-GLI2* and the *DYNLL1-GLI2* fusions in other sex cord-stromal tumors by RT-PCR and FISH, and across 33 common cancer types from The Cancer Genome Atlas (TCGA) retrieved from the Tumor Fusion Gene Data Portal^28^. Whilst the *FHL2-GLI2* and *DYNLL1-GLI2* rearrangements were present in 17/26 (65%) and 1/26 (4%) of SSTs, respectively, none of the other ovarian sex cord-stromal tumors tested by RT-PCR, namely adult-type granulosa cell tumors (*n* = 28), fibromas, fibrothecomas and thecomas (*n* = 14) and Sertoli-Leydig cell tumors (SLCTs, *n* = 6), and none of the 9950 tumors of 33 cancer types from TCGA^28^ harbored the *FHL2-GLI2* or *DYNLL1-GLI2* fusion genes (Fig. 1h, i, Supplementary Fig. 4, Supplementary Table 4). In addition, no *GLI2* break-apart signals/rearrangements were found using FISH in other sex cord-stromal tumors (*n* = 9; Supplementary Fig. 5, Supplementary Table 4). Taken together, these findings provide evidence to suggest that *FHL2-GLI2* and *DYNLL1-GLI2* fusions might be pathognomonic for SSTs of the ovary.

Consistent with the RNA-sequencing and RT-PCR findings, RNA in situ hybridization (RNA-ISH) demonstrated mRNA expression of the specific forms of the chimeric *FHL2-GLI2* fusion gene in the SSTs harboring each of the two *FHL2-GLI2* fusion genes (Fig. 1f, Supplementary Fig. 6, Supplementary Table 4). In addition, immunohistochemistry with anti-GLI2 antibodies revealed moderate to high levels of GLI2 protein expression in SSTs with and without the *FHL2-GLI2* fusion (Supplementary Figs. 2 and 4), respectively, supporting the notion that SSTs may be a disease driven by activation of GLI2.

### FHL2-GLI2 expression results in oncogenic properties

**Fig. 2:**
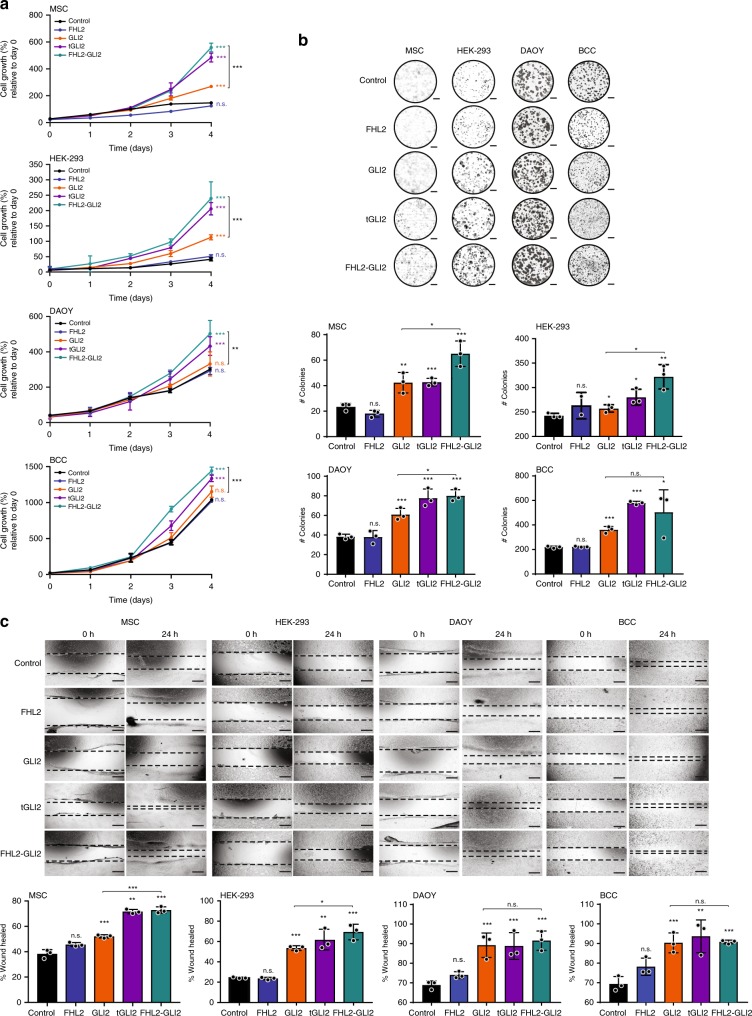
FHL2-GLI2 expression results in the acquisition of oncogenic properties in vitro. **a** Cell titer blue proliferation assay of immortalized mesenchymal stem cells (MSCs), HEK-293, medulloblastoma (DAOY), and human basal cell carcinoma (BCC) cells stably expressing empty vector (control), wild-type FHL2 (FHL2), wild-type GLI2 (GLI2), truncated GLI2 (tGLI2), or the FHL2-GLI2 fusion. **b** Representative images of colony formation assay of MSCs, HEK-293, DAOY, and BCC cells stably expressing control, FHL2, GLI2, tGLI2, or FHL2-GLI2 (scale bars, 5 mm; top). Quantification of the number of colonies/well compared to control (bottom). **c** Wound healing assay of MSCs, HEK-293, DAOY, and BCC cells stably expressing control, FHL2, GLI2, tGLI2 or FHL2-GLI2. The migratory effects/wound area was assessed at 0 and 24 h (Scale bar, 500 μm; top) and quantified (bottom). In **a**–**c**, data are representative of at least three independent experiments. Error bars, s.d. of mean; n.s., not significant; *P < 0.05, **P *<* 0.01, ***P < 0.001; two-tailed unpaired *t*-test.

Based on the known biological roles of FHL2 and GLI2, we hypothesized that cells expressing the FHL2-GLI2 fusion would exhibit oncogenic behavior through overexpression of the DNA binding and activation domains of GLI2 coupled with the loss of its repression domain. The *FHL2-GLI2* fusion gene encompassing exons 8–13 of *GLI2*, recurrently detected in SSTs, as well as truncated (t) *GLI2* lacking the repression domain were evaluated in multiple cell models, including immortalized mesenchymal stem cells (MSCs), HEK-293 cells, and in cells derived from cancers with known deregulation of the SHH pathway^22,29,30^, namely medulloblastoma (DAOY) and basal cell carcinoma (BCC) cells. MSC, HEK-293, DAOY, and BCC cells stably expressing tGLI2 or the FHL2-GLI2 fusion displayed a significant increase in proliferation and colony formation as compared to the same cells expressing empty vector, wild-type FHL2, or wild-type GLI2 (Fig. 2a, b). In addition, a scratch assay analysis revealed increased migration of MSCs, HEK-293, DAOY, and BCC cells upon stable expression of tGLI2 and FHL2-GLI2 as compared to cells expressing empty vector, wild-type FHL2 or wild-type GLI2 (Fig. 2c). Akin to the partners involved in oncogenic fusion genes, an increase in proliferation, colony formation and migration in cells transduced with wild-type GLI2 was observed, however, cells harboring tGLI2 or the FHL2-GLI2 fusion consistently significantly exhibited a more overt phenotype (Fig. 2a–c).

### FHL2-GLI2 expression results in SST phenotypic features

**Fig. 3:**
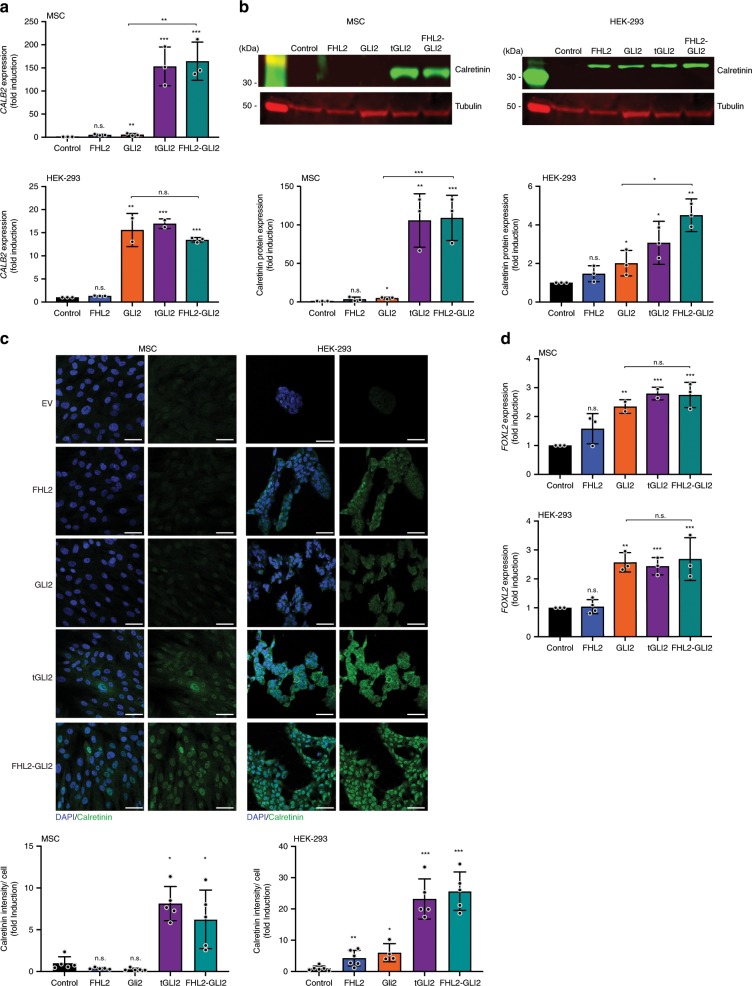
FHL2-GLI2 expression results in the expression of sex cord markers in vitro. **a** Quantitative assessment of *CALB2* transcripts in immortalized mesenchymal stem cells (MSCs) and HEK-293 cells stably expressing empty vector (control), wild-type FHL2 (FHL2), wild-type GLI2 (GLI2), truncated GLI2 (tGLI2), or the FHL2-GLI2 fusion. Expression levels were normalized to *GAPDH* expression, and comparisons of mRNA expression levels were performed relative to control. **b** Representative western blot analysis of calretinin protein levels in MSCs and HEK-293 cells stably expressing control, FHL2, GLI2, tGLI2, or FHL2-GLI2. Tubulin was used as protein loading control. Quantification (bottom) of protein levels as compared to control. **c** Representative confocal micrographs of immunofluorescence analysis of calretinin (green) and 4–6-diamidino-2-phenylindole (DAPI, blue) in MSCs and HEK-293 cells stably expressing control, FHL2, GLI2, tGLI2, or FHL2-GLI2 (scale bars, 50 μm). Quantification (bottom) of calretinin intensity/cell relative to control. **d** Quantitative assessment of *FOXL2* transcripts in MSCs and HEK-293 cells stably expressing control, FHL2, GLI2, tGLI2, or FHL2-GLI2. Expression levels were normalized to *GAPDH* expression, and comparisons of mRNA expression levels were performed relative to control. In **a**–**d**, data are representative of at least three independent experiments. Error bars, s.d. of mean; n.s., not significant; *P < 0.05, **P *<* 0.01, ***P < 0.001; two-tailed unpaired *t*-test.

We next sought to define whether the expression of the FHL2-GLI2 fusion would result in the acquisition of phenotypic features of SSTs in the cell models tested. Calretinin, a calcium-binding protein encoded by the *CALB2* gene, is primarily expressed in specific subtypes of neuronal tissue, but has also been found to be present in non-neoplastic human ovaries and sex cord-stromal tumors^31^. Specifically, calretinin is expressed in theca interna, hilus and stromal cells^31^, and has proven a useful marker in the diagnosis of sex cord-stromal tumors^32^. Based on this understanding, we posited that cells expressing the fusion FHL2-GLI2 would adopt an SST-like phenotype and express calretinin. We observed significantly higher levels of *CALB2* transcripts in MSC and HEK-293 cells stably expressing tGLI2 or the FHL2-GLI2 fusion as compared to empty vector controls or cells expressing wild-type FHL2 or wild-type GLI2 (Fig. 3a). These findings were confirmed at the protein level; western blot and immunofluorescence analyses demonstrated increased levels of calretinin in MSC and HEK-293 cells expressing tGLI2 or the fusion gene FHL2-GLI2 than in controls (Fig. 3b, c). In addition, we tested FOXL2, a highly specific marker of ovarian sex cord-stromal tumors^10^, and also found *FOXL2* gene expression levels to be higher in cells transduced with tGLI2 and the FHL2-GLI2 fusion gene than in cells expressing empty vector controls, wild-type FHL2 or GLI2 alone (Fig. 3d), providing further evidence that the presence of the *FHL2-GLI2* fusion gene results in the acquisition of some of the cardinal phenotypic characteristics of SSTs.

### FHL2-GLI2 expression results in SHH pathway activation

**Fig. 4:**
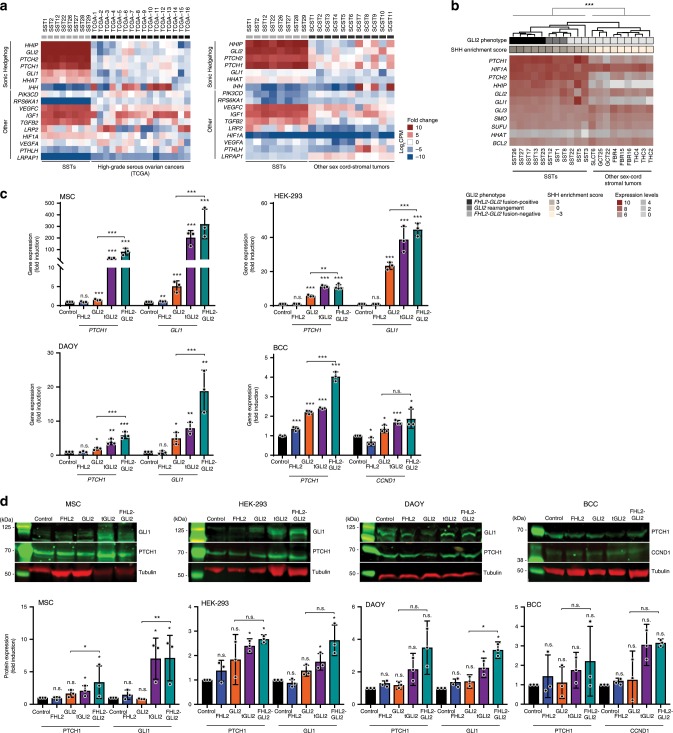
Expression of the FHL2-GLI2 fusion results in the activation of the Sonic Hedgehog pathway. **a** Differential gene expression analysis of human sclerosing stromal tumors (SSTs) subjected to RNA-sequencing (*n* = 8, this study) and high-grade serous ovarian carcinomas (*n* = 16; The Cancer Genome Atlas) and other sex cord-stromal tumors (SCSTs, *n* = 11; this study). Gene expression fold-change is color-coded according to the legend. Only genes significantly differentially expressed (*P* < 0.05; two-tailed unpaired *t*-test) are shown. CPM, count per million. **b** Expression levels of Sonic Hedgehog (SHH) pathway genes in human SSTs (*n* = 11) and other sex-cord stromal tumors (*n* = 9) as defined using NanoString. Expression levels and SHH enrichment scores are color-coded according to the legends. ***P < 0.001, Wilcoxon rank test. Hierarchical clustering was performed using complete linkage and Euclidian distance. **c** Quantitative assessment of the Sonic Hedgehog pathway *PTCH1* and *GLI1* transcripts in immortalized mesenchymal stem cells (MSCs), HEK-293 and medulloblastoma (DAOY) cells and of *PTCH1* and *CCND1* transcripts in human basal cell carcinoma (BCC) cells stably expressing empty vector (control), wild-type FHL2 (FHL2), wild-type GLI2 (GLI2), truncated GLI2 (tGLI2), or FHL2-GLI2. Expression levels were normalized to *GAPDH* expression, and comparisons of mRNA expression levels were performed relative to control. **d** Representative western blot analysis of PTCH1 and GLI1 protein expression in MSC, HEK-293 and DAOY cells and of PTCH1 and CCND1 in BCC cells stably expressing control, FHL2, GLI2, tGLI2, or FHL2-GLI2. Tubulin was used as protein loading control. Quantification (below) of protein levels as compared to control. In **c**–**d**, data are representative of at least three independent experiments. Error bars, s.d. of mean; n.s., not significant; *P < 0.05, **P *<* 0.01, ***P < 0.001; two-tailed unpaired *t*-test.

GLI2 has been reported to play pivotal roles in the regulation of the SHH signaling pathway^22–24^. To define whether the *FHL2-GLI2* fusion gene would result in activation of this pathway in SSTs, we performed a differential gene expression analysis between the eight human SSTs subjected to RNA-sequencing, common-type high-grade serous ovarian carcinomas derived from TCGA^11^ (*n* = 16) and other sex cord-stromal tumors (*n* = 11). This analysis revealed an enrichment of SHH pathway genes, including *GLI1*, *GLI2*, *HHIP*, *PTCH1*, and *PTCH2*, in SSTs as compared to high-grade serous ovarian cancers and other sex cord-stromal tumors (Fig. 4a). To determine specifically whether SHH target genes, including *PTCH1, PTCH2, HIF1A, HHIP, HHAT, GLI1/2/3, SMO*, and *SUFU*, are co-expressed with the *FHL2-GLI2* fusion gene in SSTs, we subjected SSTs (*n* = 11) and other ovarian sex cord-stromal tumors (*n* = 9; thecomas, *n* = 3; fibromas, *n* = 3; GCTs *n* = 2; and SLCT, *n* = 1) to NanoString gene expression analysis. The SHH GLI2 target genes tested were found to be co-expressed with the *FHL2-GLI2* fusion gene. In addition, we observed that the SHH pathway genes were expressed also in SSTs with other *GLI2* rearrangements, as well as in SSTs lacking *GLI2* rearrangements based on the methods employed. Regardless of the presence and type of *GLI2* rearrangements, SSTs were found to display significantly higher SHH pathway enrichment scores than other sex cord-stromal tumors (*p* < 0.001, Wilcoxon rank test; Fig. 4b, Supplementary Fig. 7).

Given the evidence that the SHH pathway activation would potentially mediate the oncogenic properties of the *FHL2-GLI2* fusion gene in human SSTs, we investigated the impact of the FHL2-GLI2 fusion expression on this pathway in our in vitro models. Consistent with the observations derived from the analysis of human SSTs, stable expression of tGLI2 and the FHL2-GLI2 fusion in MSC, HEK-293, DAOY, and BCC cells resulted in a significant increase in the expression levels of the SHH receptor *PTCH1* and/or *GLI1*/*CCND1* as compared to empty vector, wild-type FHL2 and wild-type GLI2 (Fig. 4c). For BCCs, given the very low endogenous levels of *GLI1* gene/protein expression, *CCND1* rather than *GLI1* was assessed. Again, increased *PTCH1* and *GLI1/CCND1* gene expression was also observed upon wild-type GLI2 expression, however at levels significantly lower than in tGLI2 and FHL2-GLI2 cells, both of which harbor GLI2 lacking the repression domain (Fig. 4c). This finding was further supported by quantitative western blot analyses, which demonstrated increased levels of PTCH1 and GLI1 or CCND1 proteins upon tGLI2 and FHL2-GLI2 expression in all four cell models, although the increases in PTCH1 were not significant in the DAOY and BCC cells (Fig. 4d).

These findings in human SSTs and in vitro models provide evidence to suggest that the oncogenic behavior of SSTs can at least in part be explained by activation of the SHH pathway, and the potential of *GLI2* rearrangements to serve as a marker for these uncommon ovarian neoplasms.

### *FHL2-GLI2* activates the SHH pathway via *GLI2* target promoters

**Fig. 5:**
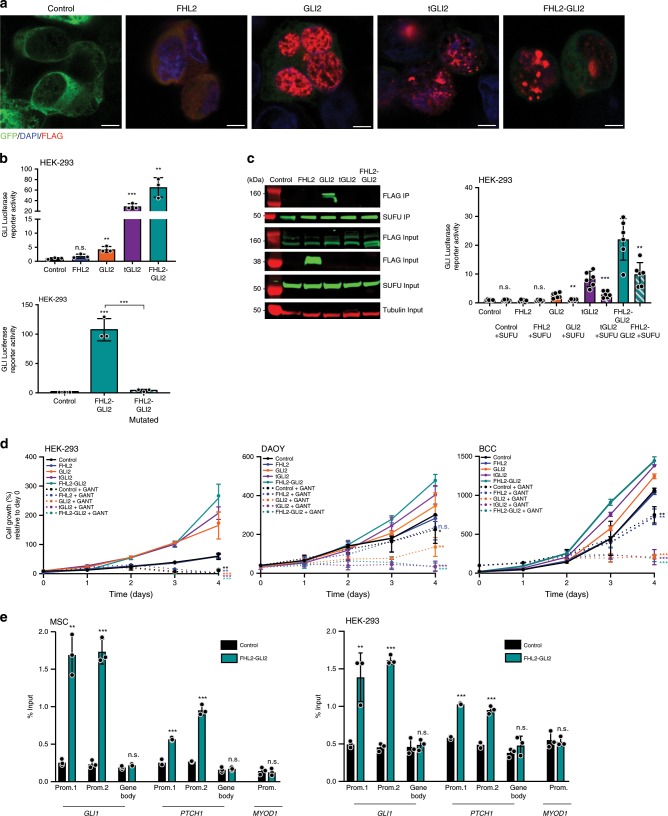
*FHL2-GLI2* activates the Sonic Hedgehog pathway through DNA binding in *GLI* target promoters. **a** Representative confocal micrographs of immunofluorescence analysis of FLAG (red), 4–6-diamidino-2-phenylindole (DAPI, blue), and GFP (green) in HEK-293 cells stably expressing empty vector (control), wild-type FHL2 (FHL2), wild-type GLI2 (GLI2), truncated GLI2 (tGLI2), or FHL2-GLI2. Scale bars, 10 μm. **b** GLI response element (GLI-RE) luciferase reporter assay of HEK-293 cells stably expressing control, FHL2, GLI2, tGLI2, or FHL2-GLI2 (top), and GLI-RE promoter activity in HEK-293 transiently transfected with control, FHL2-GLI2 and FHL2-GLI2 with R338A-K339A mutations in the Zinc Finger 5 of GLI2 required for DNA binding (FHL2-GLI2 Mutated; bottom). SV40-Renilla was used to normalize transfection efficiency. **c** Immunoprecipitation assay with SUFU antibody of HEK-293 cells stably expressing control, FHL2, GLI2, tGLI2, or FHL2-GLI2. Western blot analysis using anti-FLAG and anti-SUFU antibodies, and tubulin as loading control (left). GLI-RE promoter activity in HEK-293 stably expressing control, FHL2, GLI2, tGLI2, or FHL2-GLI2 transfected with SUFU or control (right). **d** Cell titer blue proliferation assay of HEK-293, DAOY and BCC cells stably expressing control, FHL2, GLI2, tGLI2, or FHL2-GLI2 treated with 20 µM GANT61 or vehicle control (DMSO). GANT, GANT61. **e** FLAG Chromatin Immunoprecipitation (ChIP) assay of GLI1 and PTCH1 promoters (promoter 1 and 2) in MSC and HEK-293 cells stably expressing either control or FHL2-GLI2. GLI1 and PTCH1 gene body and MYOD1, gene promoters not under GLI regulation, were used as negative controls. In **b**–**d**, data are representative of at least three independent experiments. Error bars, s.d. of mean; n.s., not significant; *P < 0.05, **P *<* 0.01, ***P < 0.001; two-tailed unpaired *t*-test.

Given the role of the SHH pathway in the oncogenesis of SSTs, we sought to define the mechanistic basis of the activation of the SHH pathway in these tumors. GLI2, a key member of the SHH pathway, has a nuclear localization where it acts as a transcription factor^33^. Immunofluorescence utilizing an anti-FLAG antibody revealed that akin to wild-type GLI2, which displayed a nuclear localization in HEK-293 cells expressing the wild-type GLI2 vector, tGLI2 and the FHL2-GLI2 fusion proteins were expressed in the nuclear compartment (Fig. 5a). These findings demonstrate that deletion of the N-terminal domain of *GLI2* does not abrogate its nuclear localization.

To test whether tGLI2 and the FHL2-GLI2 fusion proteins would activate GLI-responsive (RE) elements akin to wild-type GLI2, we performed a luciferase reporter assay with a specific GLI-RE containing GLI DNA binding sites in HEK-293, DAOY and BCC cells stably expressing empty vector, wild-type FHL2, wild-type GLI2, tGLI2, and FHL2-GLI2 (Fig. 5b, Supplementary Fig. 8). Increased activation of GLI-RE was seen in cells expressing wild-type GLI2, tGLI2, and the FHL2-GLI2 fusion but GLI-RE activation was most enhanced in tGLI2 or FHL2-GLI2 fusion gene expressing cells (Fig. 5b). Furthermore, we observed that mutation of the R338A-K339A amino acids in the Zinc Finger 5 of GLI2, which are highly conserved between all GLI family members and reported to be required for DNA binding^33^, resulted in inhibition of the capacity of the FHL2-GLI2 fusion protein to activate the SHH pathway (Fig. 5b). These data provide additional evidence that DNA binding of the FHL2-GLI2 fusion protein is necessary for the downstream activation of the SHH pathway.

We next investigated the ability of the FHL2-GLI2 fusion protein, which lacks the N-terminal binding domain, to bind to the Suppressor of fused homolog (SUFU) protein. SUFU is a known negative regulator of the SHH signaling pathway, which inhibits GLI2 through direct binding to specific regions located in the N-terminal and C-terminal regions^34^. Immunoprecipitation of SUFU revealed that both the FHL2-GLI2 fusion and tGLI2 proteins do not bind or only weakly bind to SUFU as compared to the wild-type GLI2 protein in HEK-293 cells (Fig. 5c). Luciferase experiments confirmed that SUFU overexpression is able to suppress SHH activity in HEK-293 cells expressing tGLI2 and the FHL2-GLI2 fusion protein (Fig. 5c). Inhibition of SHH activation in cells expressing tGLI2 or the FHL2-GLI2 fusion protein, which lack the N-terminal region of GLI2, likely stems from the binding of SUFU to the retained C-terminal domain of GLI2^34^. These findings were further supported by treatment of HEK-293, DAOY, and BCC cells with GANT61, a small molecule inhibitor that specifically targets the GLI1/2 DNA binding site, which resulted in decreased proliferation in all cells, but predominantly in DAOY and BCC cells expressing tGLI2 or FHL2-GLI2 (Fig. 5d). HEK-293 cells were found to be markedly sensitive to GANT61 treatment and proliferation was decreased in all vectors and conditions tested.

Finally, we sought to define whether the FHL2-GLI2 fusion protein binds to GLI target genes. We performed chromatin immunoprecipitation (ChIP) with FLAG antibody, which demonstrated the recruitment of the FHL2-GLI2 fusion protein on two different regions of both the *GLI1* and *PTCH1* promoters (promoters 1 and 2) in MSC and HEK-293 cells expressing this fusion gene (Fig. 5e)^35,36^. These results support the notion that the fusion protein binds directly to promoters of genes with GLI binding sites, thereby leading to activation of the SHH signaling pathway.

### Oncogenic properties reversed by SHH pathway inhibition

**Fig. 6:**
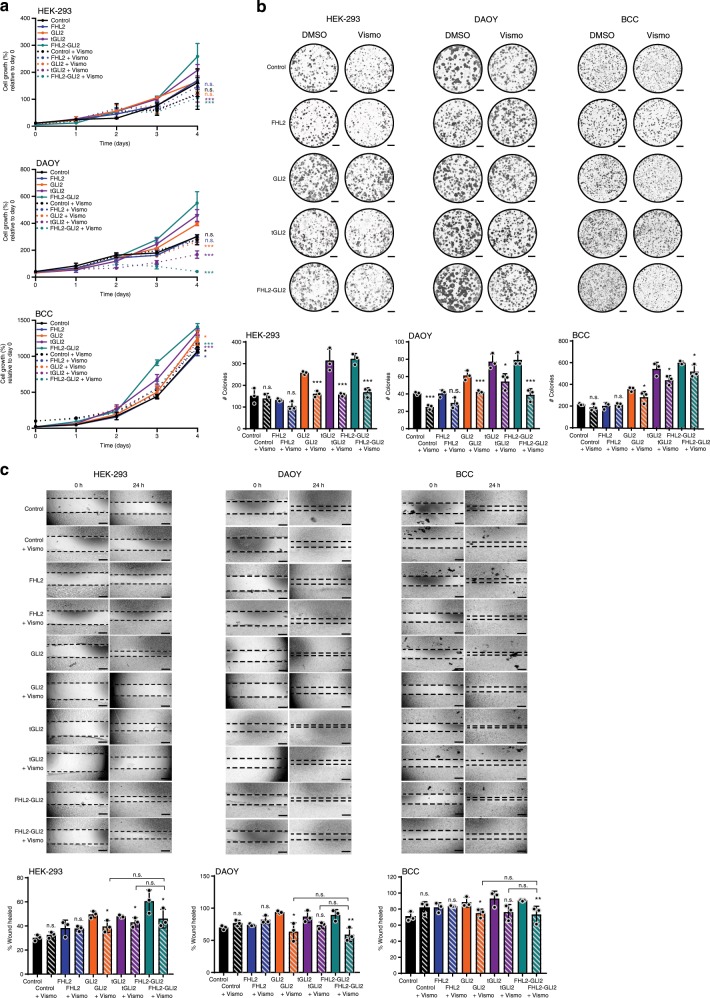
Inhibition of the Sonic Hedgehog pathway by Vismodegib reverses oncogenic properties. **a** Cell titer blue proliferation assay of immortalized mesenchymal stem cells (MSCs), HEK-293, medulloblastoma (DAOY) and human basal cell carcinoma (BCC) cells stably expressing empty vector (control), wild-type FHL2 (FHL2), wild-type GLI2 (GLI2), truncated GLI2 (tGLI2) or FHL2-GLI2 treated with 250 nM Vismodegib or vehicle control (DMSO). **b** Representative images of colony formation assay of MSC, HEK-293, DAOY, and BCC cells stably expressing control, FHL2, GLI2, tGLI2, or FHL2-GLI2 treated with Vismodegib 500 nM or vehicle control (DMSO). Scale bars, 5 mm. Quantification of the number of colonies/well compared to control (bottom). **c** Wound healing assay of MSC, HEK-293, DAOY, and BCC cells stably expressing control, FHL2, GLI2, tGLI2, or FHL2-GLI2 treated with 250 nM Vismodegib or vehicle control (DMSO). The migratory effect/wound area was assessed at 0 and 24 h and quantified compared to DMSO (bottom). Vismo, Vismodegib. Scale bars, 500 μm. In **a**–**c**, data are representative of at least three independent experiments. Error bars, s.d. of mean; n.s., not significant; *P < 0.05, **P *<* 0.01, ***P < 0.001; two-tailed unpaired *t*-test.

Antagonists targeting downstream effectors of the SHH pathway, specifically smoothened (SMO) and GLI1, have been developed as cancer therapeutics and are FDA-approved in the treatment of basal cell carcinomas^37^, tumors driven by the activation of the SHH pathway. Given the activation of the SHH pathway in human SSTs and upon FHL2-GLI2 expression in our in vitro models, we assessed whether inhibition of the SHH pathway would result in reversal of the oncogenic phenotype induced by FHL2-GLI2 expression. Upon treatment of MSC and/or HEK-293, DAOY, and BCC cells stably expressing empty vector, wild-type FHL2, wild-type GLI2, tGLI2, or the FHL2-GLI2 fusion protein with Vismodegib (Fig. 6a–c) and cyclopamine (Supplementary Fig. 9a–c), both highly specific inhibitors of SMO^37^, we observed a significant effect on proliferation and colony formation in cells expressing tGLI2 and the FHL2-GLI2 fusion protein (Fig. 6a, b, Supplementary Fig. 9a, b). We also observed a significant decrease in migration of MSC and/or HEK-293, DAOY, and BCC cells expressing tGLI2 and the FHL2-GLI2 fusion protein as compared to controls when treated with Vismodegib (Fig. 6c) or cyclopamine (Supplementary Fig. 9c).

Taken together, these results implicate the SHH pathway in the phenotype observed in cells stably transduced with the fusion *FHL2-GLI2* and provide evidence for the underlying biological basis and pathogenesis of SSTs of the ovary.

## Discussion

Here, we demonstrate that SSTs of the ovary harbor recurrent *GLI2* rearrangements, with *FHL2* being the most frequent partner gene. We further show through an analysis of other ovarian sex cord-stromal tumors and 33 different cancer types that this *FHL2-GLI2* fusion is likely pathognomonic for SSTs, providing a genotypic-phenotypic correlation in ovarian neoplasms.

GLI-transcription factors have been shown to be mediators of SHH signaling, and constitutive activation of the SHH/GLI signaling cascade has been implicated in numerous human malignancies, including skin, lung and prostate^22,38^. N-terminally truncated Gli2, unlike the full-length protein, has been reported to activate Shh target genes in transgenic mouse embryos^39^, suggesting that the modulation of the N-terminal repression domain is a mechanism of Shh signaling activation. The SHH pathway has been shown to play a prominent role in the communication between granulosa cells and developing theca cells^40,41^, and FHL2 is ubiquitously expressed in benign ovarian tissue and ovarian granulosa cells^19^. The *FHL2-GLI2* fusion gene identified here likely has an oncogenic mechanism consistent with that of several other fusion genes, where the expression of the 3′ partner gene is driven by the regulatory elements of the ubiquitously expressed 5′ partner gene^42^. In addition, the exons of GLI2 included in the *FHL2-GLI2* fusion gene lack the GLI2 repression domain but maintain its DNA binding domain. Hence, we propose a model where the *FHL2-GLI2* fusion gene results in the overexpression of a truncated GLI2 (lacking its repression domain), given that its expression is under the *FHL2* promoter, a protein ubiquitously expressed in ovarian stromal cells (i.e., the likeliest cell of origin of SSTs). Consistent with this model, the *FHL2-GLI2* fusion and t*GLI2* lacking the repression domain display nuclear localization (Fig. 5a) and result in activation of the SHH signaling pathway (Fig. 5b). In addition, the FHL2-GLI2 fusion protein was found to bind to the promoters of the GLI2 target genes *PTCH1* and *GLI1* (Fig. 5e), and was observed to require an intact GLI2 DNA binding site for the activation of the SHH pathway (Fig. 5b). Furthermore, the oncogenic effects of FHL2-GLI2 expression observed in cells could be reversed through pharmacological inhibition of the SHH pathway using three chemical inhibitors, further demonstrating the role of the GLI2-mediated SHH signaling pathway activation and dependency in cells expressing FHL2-GLI2.

We have demonstrated here that SSTs of the ovary are driven by GLI2 activation through the expression of *GLI2*-rearrangements. Whilst other rare types of ovarian neoplasms have been found to be underpinned by highly recurrent mutations, such as *FOXL2* hotspot mutations in adult-type granulosa cell tumors^9^ or *SMARCA4* loss-of-function mutations in small cell carcinomas of the ovary hypercalcemic type^12^, SSTs were found to be characterized by recurrent and likely pathognomonic *FHL2-GLI2* fusion genes. Other rare types of tumors with a relatively favorable clinical behavior have also been shown by our group and others to be driven by activating mutations and/or fusion genes. For example, adenoid cystic carcinomas of the breast are primarily driven by the *MYB-NFIB* fusion gene in ~80% of cases^43,44^; in breast adenoid cystic carcinomas lacking these fusions, *MYBL1* rearrangements and *MYB* gene amplification have been reported as alternative mechanisms resulting in MYB overexpression and activation of MYB targets^45^. Likewise, >70% of polymorphous adenocarcinomas of the salivary glands are characterized by pathognomonic *PRKD1* hotspot mutations;^46^ those with wild-type *PRKD1* have been shown to harbor *PRKD1/2/3* rearrangements^47^. Consistent with these observations, in this study, we identified a *DYNLL1-GLI2* fusion gene in an SST lacking the *FHL2-GLI2* fusion gene. Further studies are warranted to define the fusion partners of the SSTs with *GLI2* rearrangements but lacking *FHL2-GLI2* and *DYNLL1-GLI2* fusions, and to determine the mechanisms leading to GLI2 and SHH signaling pathway activation in SSTs lacking fusion genes involving *GLI2*.

This study has several limitations. Due to the retrospective multi-institutional nature of our study, follow-up information was unavailable, and the impact of the *FHL2-GLI2* fusion on patient outcome could not be assessed. We did not identify a *GLI2* rearrangement or alternative driver genetic alteration in 19% (5/26) of the SSTs studied. In addition, in three SSTs, FISH revealed the presence of *GLI2* rearrangements, however the partner genes could not be identified. Of note, GLI2 protein expression and SHH pathway activation were also observed in SSTs lacking any of the *GLI2* rearrangements identified here (Fig. 4, Supplementary Figs. 2 and 6), providing evidence to suggest that mechanisms other than the *FHL2-GLI2* and *DYNLL1-GLI2* fusion genes may lead to the constitutive activation of GLI2 in these lesions. Further studies, ideally utilizing frozen samples of SSTs with *GLI2* rearrangements other than those described in this study or lacking *GLI2* rearrangements altogether are warranted to define their driver alterations. Finally, due to the lack of representative cell line models derived from human SSTs, we established cell models using MSC, HEK-293, DAOY, and BCC cells with stable expression of the FHL2-GLI2 fusion and tGLI2. Despite HEK-293 cells not being representative of the likely cell of origin, FHL2-GLI2 expression resulted in the acquisition of phenotypic characteristics of sex cord-stromal tumors, including expression of sex cord markers and SHH pathway activation. Whilst we utilized DAOY and BCC cell lines given their dependence on the SHH pathway^22,48,49^, as expected in the setting of preexisting activation of GLI-signaling, the effects of FHL2-GLI2 expression in DAOY and BCC cell lines, albeit mostly significant, were not as overt as those observed in MSCs and HEK-293 cells.

Despite these limitations, we report on the pathognomonic fusion gene *FHL2-GLI2* in SSTs of the ovary, which results in the activation of the SHH pathway. Our findings provide evidence to suggest that *GLI2* rearrangements are likely drivers of SSTs and may serve as potential ancillary markers for the diagnosis of these tumors, and that *GLI2* should be included in panels surveying fusion genes in human cancers. Finally, our results on the SHH pathway activation in SSTs provide a potential therapeutic avenue for patients with SSTs, with FDA-approved SHH pathway inhibitors being available.

## Methods

### Subjects and samples

Following approval by the Institutional Review Boards (IRBs)/local ethics committees of the authors’ institutions, unstained tissue sections from formalin-fixed paraffin-embedded (FFPE) SSTs were retrieved from Fudan University Cancer Center’s consultation cases (Shanghai, China) and Massachusetts General Hospital (MGH, MA, USA). Patient consents were obtained following the respective IRB protocols approved by the authors’ institutions, and samples were anonymized before analysis. All tumors were centrally reviewed by four pathologists (R.B., J.S.R.-F., R.A.S. and E.O.), and 26 tumors were classified as SSTs and included in this study.

The discovery and validation series comprised 8 and 18 SSTs, respectively (Supplementary Table 1, Supplementary Fig. 1). The 8 SSTs from the discovery series were subjected to microdissection, RNA extraction and RNA-sequencing, and to microdissection of the tumor and normal tissues, DNA extraction and whole-exome (*n* = 3) or MSK-IMPACT (*n* = 5) sequencing (Fig. 1, Supplementary Table 1, Supplementary Fig. 1). FFPE-derived RNA from all microdissected SSTs were subjected to RT-PCR (*n* = 26; discovery and validation series), and 13 SSTs were subjected to fluorescence in situ hybridization (FISH; Fig. 1, Supplementary Fig. 1, Supplementary Table 4). In addition, RNA from other ovarian sex cord-stromal tumors was also subjected to RT-PCR (*n* = 48) and/or FISH (*n* = 9; Figs. 1, 4, Supplementary Fig. 1, Supplementary Table 4).

### Whole-exome and targeted massively parallel sequencing

Microdissected tumor and normal DNA from 3 SSTs was subjected to whole-exome sequencing (WES), and microdissected tumor DNA from 5 SSTs to MSK-IMPACT sequencing (Supplementary Fig. 1, Supplementary Table 1), a massively parallel sequencing assay targeting all exons and selected introns of 410 cancer-related genes^16^. Sequencing was performed at Memorial Sloan Kettering Cancer Center’s (MSKCC’s) Integrated Genomics Operation (IGO) using validated protocols^50,51^. Sequencing data were analyzed and mutations identified using validated bioinformatics methods^50,51^. Mutations affecting hotspot codons were annotated according to Chang et al.^52^. Copy number alterations (CNAs) and loss of heterozygosity (LOH) were defined using FACETS^53^ as described^50,51^. ABSOLUTE (v1.0.6)^54^ was employed to determine the cancer cell fraction (CCF) of each mutation^50,51^.

### RNA-sequencing and fusion gene identification

Eight SSTs were subjected to paired-end RNA-sequencing (Supplementary Fig. 1**;** Supplementary Table 1) using validated protocols at MSKCC’s IGO^51,55^. Read pairs supporting fusion transcripts were identified using INTEGRATE^56^, deFuse^57^ and FusionCatcher^58^, as described^51,55^. To filter out common alignment artifacts and normal transcriptional variation, we removed fusion gene and read-through candidates that were also found in a set of 297 normal samples from The Cancer Genome Atlas (TCGA)^59^. The remaining in-frame candidate fusion genes were annotated to predict their oncogenic/driver potential using OncoFuse (v1.0.9b2)^17^. The presence of identified fusion gene candidates was assessed in other cancer types using the Tumor Fusion Gene Data Portal^28^, which comprises 20,731 fusion genes across 33 cancer types (*n* = 9950; October 2019).

### Differential gene expression analysis

For differential gene expression analysis, RNA-sequencing data of 8 SSTs (discovery cohort), of 11 other sex cord-stromal tumors (this study; adult-type granulosa cell tumors, *n* = 6; juvenile-type granulosa cell tumors, *n* = 2; and Sertoli-Leydig cell tumors, *n* = 3) and of high-grade serous ovarian carcinomas derived from TCGA^11^ (*n* = 16; GDC Legacy Archive, https://portal.gdc.cancer.gov/legacy-archive/) were employed. Transcriptomic analysis was performed in the R environment using edgeR (v3.24.3)^60^. Genes with less than 0.2 count per million (CPM) were labeled as poorly expressed across all libraries and removed from further analyses. Raw counts were normalized using an upper quartile of M-values approach^61^, and dispersion estimates over all genes were carried out with the quantile-adjusted conditional maximum likelihood method^62^. Resulting *p*-values were corrected for multiple testing using the Benjamini-Hochberg false discovery rate^63^. Furthermore, Reads Per Kilobase Million (RPKM) values were estimated from raw counts normalizing for sequencing depth and adjusting for length of genes.

### *FHL2-GLI2* fluorescence in situ hybridization

*FHL2-GLI2* fusion gene assessment by FISH was performed using validated protocols at MSKCC’s Molecular Cytogenetics Core^64^. Probes for detection of the *FHL2-GLI2* fusion gene consisted of a three-color probe mix, composed of bacterial artificial chromosomes (BACs) mapping to 5′ *GLI2* (orange), 3′ *GLI2* (red), and 5′ *FHL2* (green). To screen for the presence of *GLI2* rearrangements in SSTs lacking *FHL2-GLI2/DYNLL1-GLI2* fusions by RNA-sequencing and/or RT-PCR, probes for the detection of the *GLI2* gene consisted of a dual-color probe mix, composed of the same BACs as above mapping to 5′ *GLI2* (yellow) and 3′ *GLI2* (green). At least 10 images per tumor region were captured and at least 50 non-overlapping interphase nuclei with well-delineated contours were analyzed for the presence of the *FHL2-GLI2* fusion or *GLI2* rearrangements. SSTs were considered positive for the *FHL2-GLI2* fusion gene or for *GLI2* rearrangements if >15% of the cells displayed at least one 5’*FHL2–*3’*GLI2* fusion or 5’*GLI2* or 3’*GLI2* signal.

### NanoString gene expression analysis

A NanoString nCounter gene expression assay using a custom CodeSet panel of SSH pathway genes was performed according to the manufacturer’s instructions (NanoString Technologies). NanoString nCounter data were analyzed using the R Bioconductor package NanoStringDiff ^65^, an approach which facilitates analysis of nCounter data using a negative binomial generalized linear model (GLM) with differential expression analysis performed using model-based linear contrasts, and associated inference based on likelihood ratio tests. The negative binomial GLM incorporates normalization factors for positive controls, background noise and the 5 housekeeping genes. Single sample Gene Set Enrichment (ssGSEA) analysis^66^ was used to define the Sonic Hedgehog (SHH) enrichment score for each sample. Hierarchical cluster analysis of gene expression data was performed using complete linkage and Euclidian distance.

### Single-molecule RNA in situ hybridization

RNA in situ hybridization (RNA-ISH) experiments were performed using RNAscope, according to the manufacturer’s instructions^67^. Paired double-Z oligonucleotide probes were designed against target RNA: BA-Hs-GLI2–3EJ (Cat no. 801211, NM_005270.4, 3zz pair, nt 850–1221), BA-Hs-FHL2-GLI2-FJ (Cat no. 801221, chr2:105984027 > chr2:121732551, 1zz pair, nt 53–90), BA-Hs-FHL2-GLI2-FJ-O1 (Cat no. 801231, chr2:106002818 > chr2:121729517, 1zz pair, nt 57–94) (Supplementary Table 3). The BaseScope Reagent Kit (Advanced Cell Diagnostics) was used and FFPE tissue sections were prepared according to the manufacturer’s instructions. Each sample was quality controlled for RNA integrity with a 1zz probe specific to the housekeeping gene *PPIB*. Negative control background staining was evaluated using a 1zz probe specific to the bacterial *dapB* gene.

### Reverse transcription PCR (RT-PCR) for fusion validation

Total RNA was reverse-transcribed using SuperScript VILO Master Mix (Life Technologies; Thermo Fisher Scientific), according to the manufacturer’s instructions. PCR amplification of 10 ng of cDNA was performed using specific primer sets designed based for each fusion gene and each breakpoint (Supplementary Table 3)^51^. PCR fragments were purified (ExoSAP-IT, Affymetrix) and sequenced on an ABI 3730 capillary sequencer using the ABI BigDye Terminator chemistry (v3.1, Life Technologies) according to the manufacturer’s protocol. Sequences of the forward and reverse strands were analyzed using MacVector software (MacVector, Inc). All analyses were performed in duplicate.

### GLI2 and Ki67 immunohistochemistry

Representative 4 μm FFPE SST tissue sections on Superfrost Plus slides (Thermo Fisher Scientific) were subjected to antigen retrieval using Epitope Retrieval 1 (ER1) for 30 min. Double immunohistochemical staining for GLI2 and Ki67 was performed using the ChromoPlex 1 Dual Detection for BOND kit (Leica, DS9477) with a mouse monoclonal antibody against GLI2 (clone OTl1G1, TA804601, Origene) at a dilution of 1:500 (2 μg/ml) stained with red chromogen and a mouse monoclonal against Ki67 (MIB-1, Dako) at a dilution of 1:400 (46 μg/ml) stained with 3,3′-diaminobenzidine tetrahydrochloride (DAB) for 30 min and visualized on a Leica Bond-III autostainer (Leica, Buffalo Grove, III). Negative controls (omission of the primary antibody and substitution of the primary antibody with IgG-matched control) and positive controls (formalin-fixed, paraffin-embedded pellets of cell lines stably expressing GLI2) were included in each slide run, as described^46^.

### Cell lines

Immortalized mesenchymal stem cells (MSCs; ASC52telo, ATCC), HEK-293 cells (ATCC), DAOY medulloblastoma cells (ATCC), and Human Basal Cell Carcinoma (BCC; CELPROGEN) primary cells were authenticated using short tandem repeat profiling at MSKCC IGO and tested for mycoplasma using the PCR-based Universal Mycoplasma Detection kit (ATCC). MSCs were cultured in Mesenchymal Stem Cell Growth Medium (Lonza). HEK-293 cells were cultured in Dulbecco’s modified Eagle’s medium (DMEM) high glucose supplemented with 10% fetal bovine serum (FBS) and 1% penicillin/streptomycin. DAOY cells were cultured in Eagle’s Minimum Essential Medium (EMEM) supplemented with 10% fetal bovine serum (FBS) and 1% penicillin/streptomycin. BCC cells were cultured in Human Basal Cell Carcinoma Cell Line Complete Media (CELPROGEN). All cell lines were maintained in a 5% CO_2_ atmosphere at 37 °C.

### Generation of stable cell lines

Human wild-type *FHL2* (EX-M0686-Lv102), wild-type *GLI2* (EX-Y4001-Lv102), truncated *GLI2* lacking the N-terminal domain constructed from *GLI2* (EX-Y4001-Lv102), *FHL2-GLI2* (CS-Y4001-Lv102–01) ORF cDNA clones with N-terminal FLAG tag were purchased from GeneCopoeia. Individual FLAG-tagged cDNAs were amplified and cloned into pCDH-CMV-MCS-EF1α-GFP-T2A-Puro cDNA Dual Promoter Cloning and Expression Lentivector (CD513B-1, System Biosciences) (primers, see Supplementary Table 5). Empty vector (EV) was used as a control. Lentiviral particles were produced in MSKCC’s Gene Editing and Screening Core Facility. MSC, HEK-293, DAOY and BCC cells were infected for 24 h and then selected for 14 days in puromycin (2 µg/ml; ThermoFisher Scientific). Transduction efficiency was confirmed by quantitative RT-PCR and western blot.

### *FHL2-GLI2* mutagenesis

R338A-K339A mutations were introduced into the *FHL2-GLI2* sequence using the Q5 Mutagenesis Kit (New England Biolabs) following the manufacturer’s instructions and transformed in NEB Stable E.Coli to avoid unspecific recombination of the plasmid. The mutagenesis primers were designed using the NEBaseChanger (Forward: 5-CAGCTCTCTCgcggcgCATGTGAAAAC-3; Reverse: 5-GGGTCTGTGTATCTCTTG-3) and the mutations validated using Sanger sequencing (primer: 5-ggagcagaagcccttcaa-3).

### Luciferase assays

Stable HEK-293, DAOY, and BCC cell lines expressing empty vector, wild-type FHL2, wild-type GLI2, truncated GLI2 (tGLI2), and FHL2-GLI2 were plated in 96 well plates (25,000 cells/well) and transfected with lipofectamine 3000 (Thermo Fisher Scientific) following the manufacturer’s instructions. Gli-Luc reporter pGL4[luc2P/Gli-RE/Hygro] (90 ng; Promega) and the transfection efficiency control Renilla-SV40 (10 ng; Promega) were added, incubated for 48 h and the luciferase levels assessed using the Dual luciferase kit (Dual luciferase kit; Promega) and read on the Glomax96 luminometer following the manufacturer’s recommendations. SUFU overexpressing plasmid (NM_016169, Human Tagged ORF Clone from Origene) was co-transfected where indicated. HEK-293 cells were transiently co-transfected with CD513B1_FHL2-GLI2 or CD513B1_FHL2-GLI2_Mutated or CD513B1 empty vector were indicated.

### Quantitative RT-PCR

Total RNA was extracted from cell lines using the RNeasy Mini Kit (Qiagen) and tumors using RNeasy FFPE kit (Qiagen) and reverse-transcribed into cDNA using SuperScript VILO Master Mix (ThermoFisher) according to the manufacturer’s instructions. Quantitative TaqMan RT-PCR was performed for *FHL2* (Hs00991866), *GLI2* (Hs00171790), *CALB2* (Hs00242372), *GLI1* (Hs00171790), *PTCH1* (Hs00181117), *FOXL2* (Hs00846401), and *CCND1* (Hs00765553_m1) using the StepOnePlus Real-Time PCR System (Applied Biosystems; ThermoFisher). All experiments were performed in triplicate, and expression data were normalized to *GAPDH* (Hs02786624), as described^68^.

### Crosslinked chromatin immunoprecipitation (ChIP)

The ChIP assay was performed as described^69^. Briefly, ten million MSC and HEK-293 cells expressing either empty vector or FHL2-GLI2 were used per ChIP. Chromatin was cross-linked by the addition of 1% formaldehyde (Sigma-Aldrich), and DNA was sheared to 200–500 bp fragments by sonication (Bioruptor, Diagenode). For ChIP, 50 µl ANTI-FLAG M2 Affinity Gel (Sigma) were added and incubated at 4 °C overnight. Beads were incubated with elution buffer (50 mM Tris pH 8.0, 10 mM EDTA, 1% SDS, 0.1 M NaHCO_3_, and 250 mM NaCl) at 65 °C overnight to elute the immune complexes. ChIP and input DNA were purified using a QIAquick PCR purification kit (Qiagen). qPCR was performed using PowerUp SYBR Green Master Mix (Applied Biosystems). Primers for *GLI1*, *PTCH1*, and *MYOD1* are listed in Supplementary Table 6.

### Immunoprecipitation

For SUFU immunoprecipitation, 10 million HEK-293 cells were treated with MG-132 10 μM for 6 h. Protein was extracted with IP buffer (1% NP-40, 50 mM Tris-HCl pH 8.0, 150 mM NaCl + Halt Protease Inhibitor Cocktail; Thermo Fisher Scientific), and 500 µg of protein lysate were preincubated for 1 h at 4 °C with 4 µl of SUFU antibody (C81H7; Rabbit mAb #2522; Cell Signaling Technology) in 500 µl IP buffer. Twenty five microliter of Protein G Dynabeads (Thermo Fisher Scientific) were added and incubated overnight at 4 °C. After washing the beads 3 times in IP Buffer and resuspension in 20 µl of 1× NuPAGE LDS buffer, the beads were heated at 90 °C for 10 min and the supernatant was recovered and run on a western blot as described below. The membrane was blotted with anti-FLAG M2, SUFU and tubulin antibodies (1:1000). Total protein extractions were also tested as loading control.

### Western blotting

Standard western blotting was conducted^70^. Cell pellets were solubilized in RIPA buffer with 5% Halt Protease and Phosphatase Inhibitor Cocktail and 2% 0.5 mM EDTA (all Thermo Fisher Scientific). Extracts were centrifuged and the supernatant was then recovered for SDS-PAGE. Primary antibodies against calretinin (Santa Cruz, SC-365956, 1:500), PTCH1 (Abcam, ab53715, 1:1000), GLI1 (Abcam, ab134906, 1:500), GLI2 (Origene, TA804601, 1:500), Cyclin D1 (Cell Signaling, 92G2, #2978, 1:1000), FLAG M2 (Sigma, F1804, 1:1000), SUFU (Cell Signaling, #2522 s, 1:1000), and tubulin (Cell Signaling, DM1A, 1:1000). Conjugated secondary antibodies anti-rabbit (LI-COR, 926–68073, 1:10,000) and anti-mouse (LI-COR, 926–32212, 1:10,000) were used and detected using the Odyssey Infrared Imaging System (LI-COR Biosciences), as described (Supplementary Figs. 10–13)^70^. Quantification and analysis were performed using ImageJ. Experiments were performed in triplicate.

### Proliferation assay

Cells were seeded in 96-well plates (1000 cells/well; *n* = 6 for each cell line and condition). Proliferation rate was assessed using the Cell Titer-Blue Cell Viability Assay (Promega)^46,50^. Absorbance was detected with 560 nm excitation and 590 nm emission using a Victor X4 Multimode Plate Reader (PerkinElmer). Experiments were performed in triplicate.

### Scratch wound healing assay

Cells were seeded in 24-well plates at 90–95% confluence. On the following day, a scratch was made on the cell monolayer using a 1 ml pipette tip across the center of the well. Phase-contrast images were obtained at 0 and 24 h following scratch wounding, using an EVOS XL Core Microscope (ThermoFisher Scientific), the wound area was measured using ImageJ, and the data analyzed to determine percent of wound closure, as described^50^. Experiments for each condition were performed in triplicate in at least 3 independent experiments.

### Colony formation assay

Cells were seeded in 6-well plates (500–100 cells/well). After 10 days, cells were fixed and stained using the sulforhodamine B (SRB) protocol, as described^46,50^. In brief, cells were fixed at 4 °C for 1 h using Trichloroacetic acid (TCA) (Sigma), washed then stained on a rocking platform for 30 min with 0.04% SRB in 1% acetic acid. Plates were imaged using Zeiss Observer Z1, ×5/0.5NA objective and Hamamatsu Flash V3 cMOS camera. Motorized stage was calibrated to tile-image the entirety of each well. Stitched images were analyzed in ImageJ/FIJI using customized macro script. Gaussian and median filters were used to blur the image before colonies were detected by intensity threshold. Touching colonies were separated using Watershed algorithm. The number of colonies and the area of each were analyzed. Quantification of the number of colonies per well and colony size was performed using ImageJ. Experiments were performed in triplicate.

### Immunofluorescence

MSCs HEK-293 cells were grown on glass coverslips and fixed with 4% formaldehyde (ThermoFisher Scientific), blocked in 10% normal Goat Serum (Vector Laboratories, S-1000) then incubated with primary antibody against Calretinin (Santa Cruz, sc-565956, 1:100) followed by Alexa Fluor-conjugated secondary antibody Alexa 647 goat anti-mouse (Life Technologies, A21236, 1:500). For GLI2 nuclear localization studies, HEK-293 cells in slide chambers were fixed with ice cold solution 1:1 acetone:methanol for 15 min at −20 °C. After washing 3 times with PBS the samples were blocked with blocking solution (PBS; 5% goat serum). The samples were incubated overnight at 4 °C with anti-FLAG M2 antibody (Sigma, F-1804, 1:200 in PBS/1% BSA), followed by Alexa 647 tagged secondary goat anti-mouse antibody (Life Technologies, A21236, 1:500). Slides were mounted using Gold Prolong antifade reagent containing DAPI (ThermoFisher Scientific, P36930). Fluorescent images were acquired at MSKCC’s Molecular Cytology Core using a TCS SP5 upright Confocal microscope (Leica Microsystems), with a ×63/1.40 oil objective, HyD hybrid detectors, and the Leica Application Suite Advanced Fluorescence (LASAF) acquisition software (Leica Microsystems). DAPI and Alexa647 were excited with 405 nm and 650 nm lasers, respectively, and imaged in the range of 410–460 nm and 660–700 nm, respectively. Linear LUT was used at full range. Images were acquired as LIF files (Leica image file format; Leica Microsystems) at a resolution of 1024 × 1024 pixels, a scaling of 0.12 micron/pixel, and an 8-bit depth and processed using Fiji and ImageJ. No post-acquisition processing was performed, besides minor adjustments of brightness and contrast, applied equally to all images. ImageJ software was used to quantify the number of pixels per cell analyzing at least five representative images (×40 field) for each condition. Calretinin images were analyzed using ImageJ/FIJI with a custom macro. The number of cells within each image were counted by segmenting the DAPI channels. After setting an appropriate threshold, total intensity of calretinin was measured for the image then normalized to the number of cells. Experiments for each condition were performed in triplicate in at least 3 independent experiments.

### Inhibitors

The smoothened inhibitors Cyclopamine (Selleckchem, S1146), Vismodegib (Cellagen tech, C4044–5) and the GLI inhibitor GANT61 (Tocris, 3191) were resuspended in DMSO and used in in vitro proliferation, colony formation and scratch wound healing assays at 5–10 µM, 250–500 nM, and 5–10 µM, respectively. Experiments were performed in triplicate.

### Statistical analysis

Statistical analysis was performed using Prism 7 (GraphPad). Student’s two-tailed *t*-test was employed for the comparison of means in parametric data. The heteroscedasticity was assessed for each comparison, and homoscedastic or heteroscedastic *t*-tests were employed as appropriate, as described^50^. Wilcoxon rank test was used for the comparison of the SHH enrichment scores. A *P* value of <0.05 was considered significant.

### Reporting summary

Further information on research design is available in the Nature Research Reporting Summary linked to this article.

## Supplementary information

Supplementary Information

Reporting Summary

## Data Availability

RNA-sequencing data have been deposited in Sequence Read Archive (SRA) with accession code PRJNA540984. WES and targeted sequencing data are available via cBioPortal for Cancer Genomics (www.cbioportal.org). The remaining data are available in the Article or Supplementary Material.
