# Supplementary material for: Identification of recurrent FHL2-GLI2 oncogenic fusion in sclerosing stromal tumors of the ovary
Source: Nat Commun. 2020 Jan 2;11:44. doi: 10.1038/s41467-019-13806-x (PMC6940380; doi:10.1038/s41467-019-13806-x)
Supplement: Supplementary file 1 — Supplementary Information [file 41467_2019_13806_MOESM1_ESM.pdf]

SUPPLEMENTARY INFORMATION

Identification of recurrent *FHL2-GLI2* oncogenic fusion in sclerosing stromal tumors of the ovary

Kim et al.

Supplementary Figures 1-13

Supplementary Tables 1-6

Supplementary Figure 1

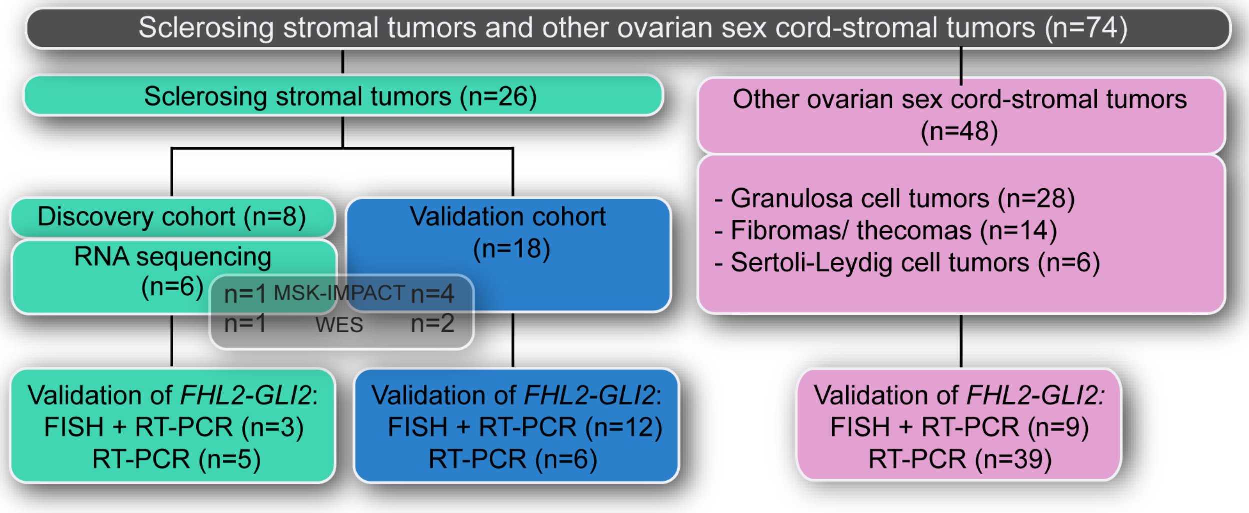

Supplementary Figure 1: Schematic representation of the tissue samples and sequencing methods employed in this study. Depiction of the discovery and validation cohorts of sclerosing stromal tumors of the ovary, and of other ovarian sex cord-stromal tumors included in this study, and the sequencing methods employed. FISH, fluorescence *in situ* hybridization; RT-PCR, reverse transcription PCR.

Supplementary Figure 2

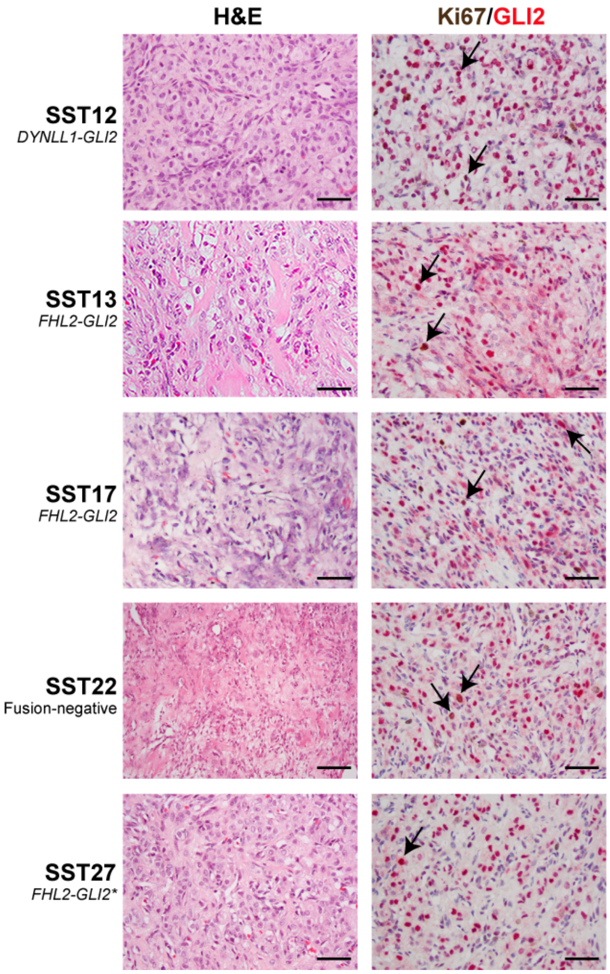

Supplementary Figure 2: Immunohistochemical analysis of GLI2 and Ki67 in sclerosing stromal tumors of the ovary. Representative micrographs of sclerosing stromal tumor sections subjected to (left) hematoxylin & eosin (H&E) staining and (right) GLI2 (red) and Ki67 (brown) immunohistochemical analysis. Note the nuclear GLI2 expression and colocalization with Ki67 (arrows). Scale bars, 20 µm. *exon 7 *GLI2* breakpoint.

Supplementary Figure 3

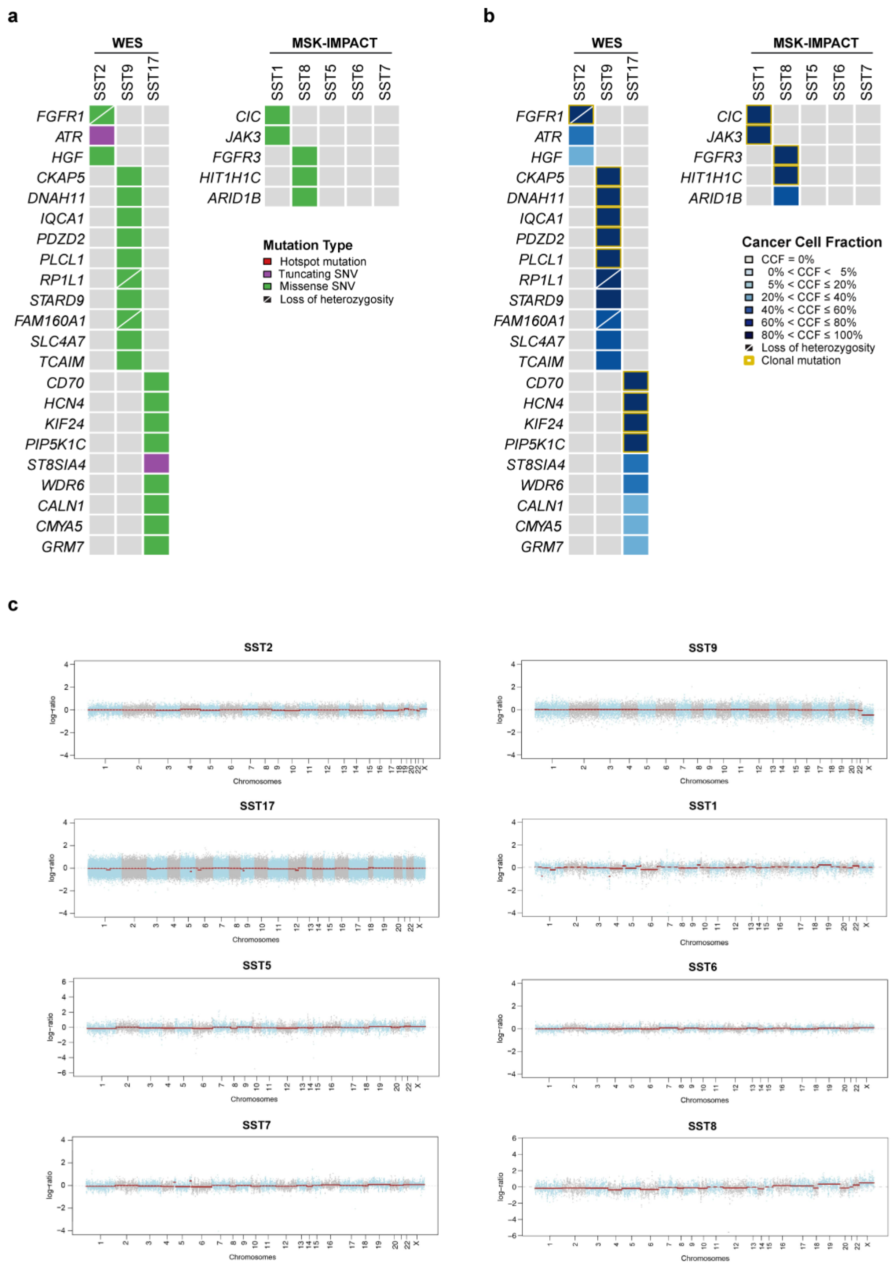

Supplementary Figure 3: **Landscape of somatic mutations and copy number alterations of sclerosing stromal tumors of the ovary.** Heatmap depicting (**a**) non-synonymous somatic mutations and (**b**) cancer cell fractions of identified mutations in sclerosing stromal tumors identified by whole-exome sequencing (WES) and by MSK-IMPACT. Mutations and cancer cell fractions are color coded according to the legend. (**c**) Copy number plots of sclerosing stromal tumors subjected to whole-exome or MSK-IMPACT sequencing. The Log_2 ratios are plotted on the y-axis according to genomic positions (x-axis). Chromosomes are depicted by alternating blue and red bands.

Supplementary Figure 4

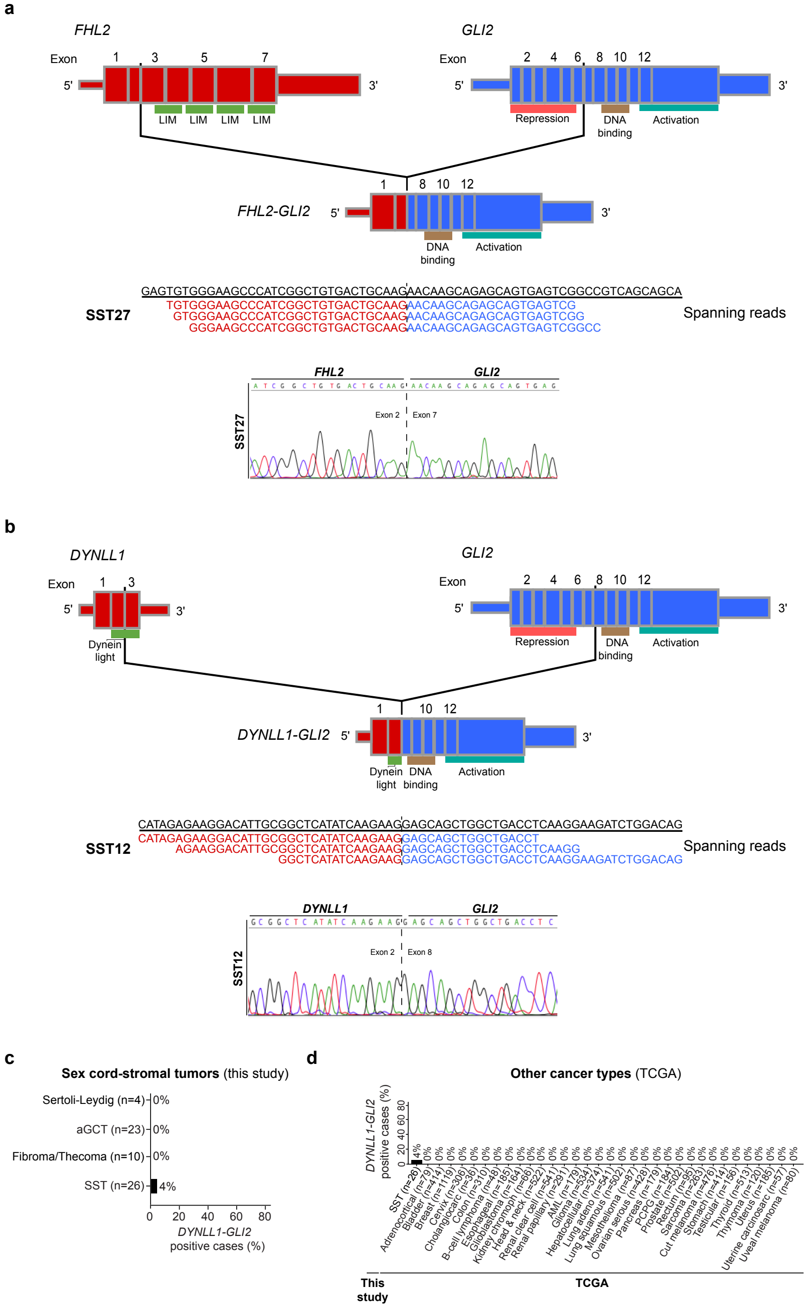

Supplementary Figure 4: Unique *FHL2-GLI2* and *DYNLL1-GLI2* fusion genes in sclerosing stromal tumors of the ovary. (**a**) Schematic representation of the *FHL2-GLI2* fusion transcript involving exon 7 of GLI2 and (**b**) of the *DYNLL1-GLI2* fusion transcript including the exons and domains involved. The breakpoint of the 5' and 3' partner genes are represented as black vertical lines (top). Spanning reads are depicted and aligned to the predicted junction sequence (middle). Representative Sanger sequencing electropherograms of the genomic *FHL2-GLI2* breakpoint (bottom). (**c**) Frequency of the *DYNLL1-GLI2* fusion in other sex cord-stromal tumors. aGCT, adult-type granulosa cell tumor. (**d**) Frequency of the *DYNLL1-GLI2* in 33 cancer types from The Cancer Genome Atlas. AML, acute myeloid leukemia; PCPG, pheochromocytoma and paraganglioma; SST, sclerosing stromal tumor.

Supplementary Figure 5

a

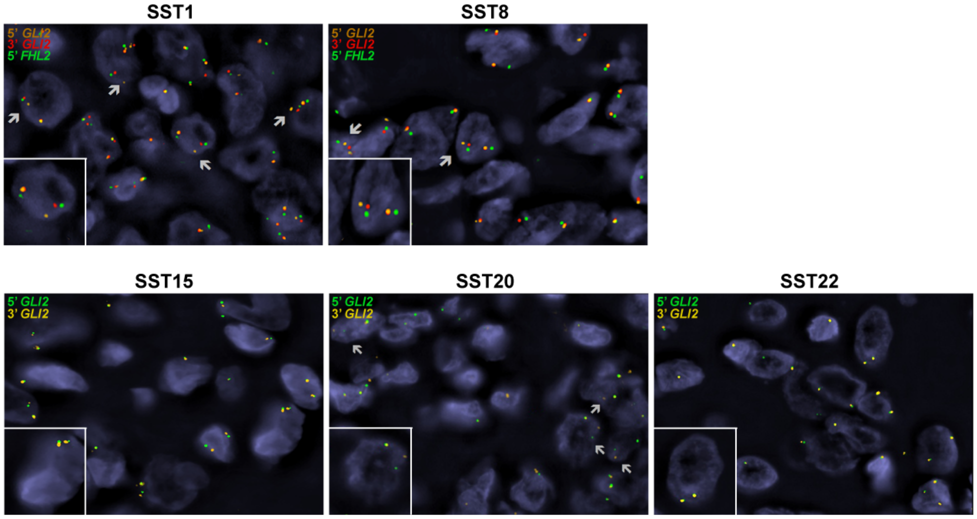

b

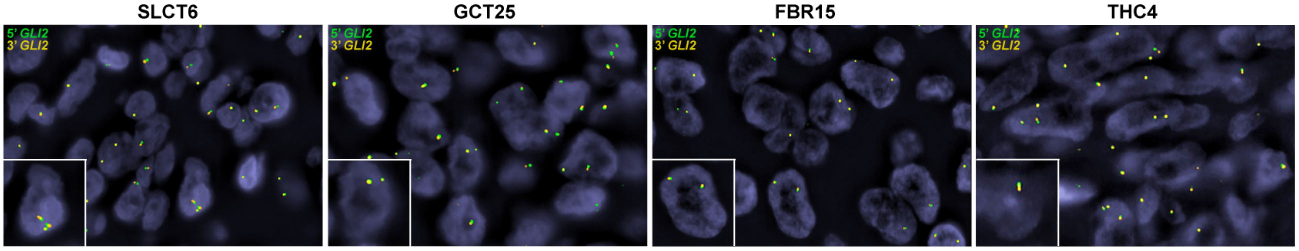

Supplementary Figure 5: Fluorescence *in situ* hybridization (FISH) analysis of sclerosing stromal tumors of the ovary and other sex cord-stromal tumors. (**a**) FISH analysis using a three-color *FHL2-GLI2* probe with 5′ *GLI2* (orange), 3′ *GLI2* (red) and 5′ *FHL2* (green) showing rearrangements in *GLI2* in SST1 and SST 8 (arrows), or a dual-color *GLI2* probe with 5′ *GLI2* (green) and 3′ *GLI2* (yellow) showing *GLI2* break-apart signals in SST 20 (arrows). No *GLI2* rearrangements were found in SST15 and SST22. (**b**) FISH analysis of other sex cord-stromal tumors using a dual-color *GLI2* probe with 5′ *GLI2* (green) and 3′ *GLI2* (yellow) showing absence of *GLI2* break-apart signals in all cases. GCT, granulosa cell tumor; FBR, fibroma; SLCT, Sertoli-Leydig cell tumor; THC, thecoma.

Supplementary Figure 6

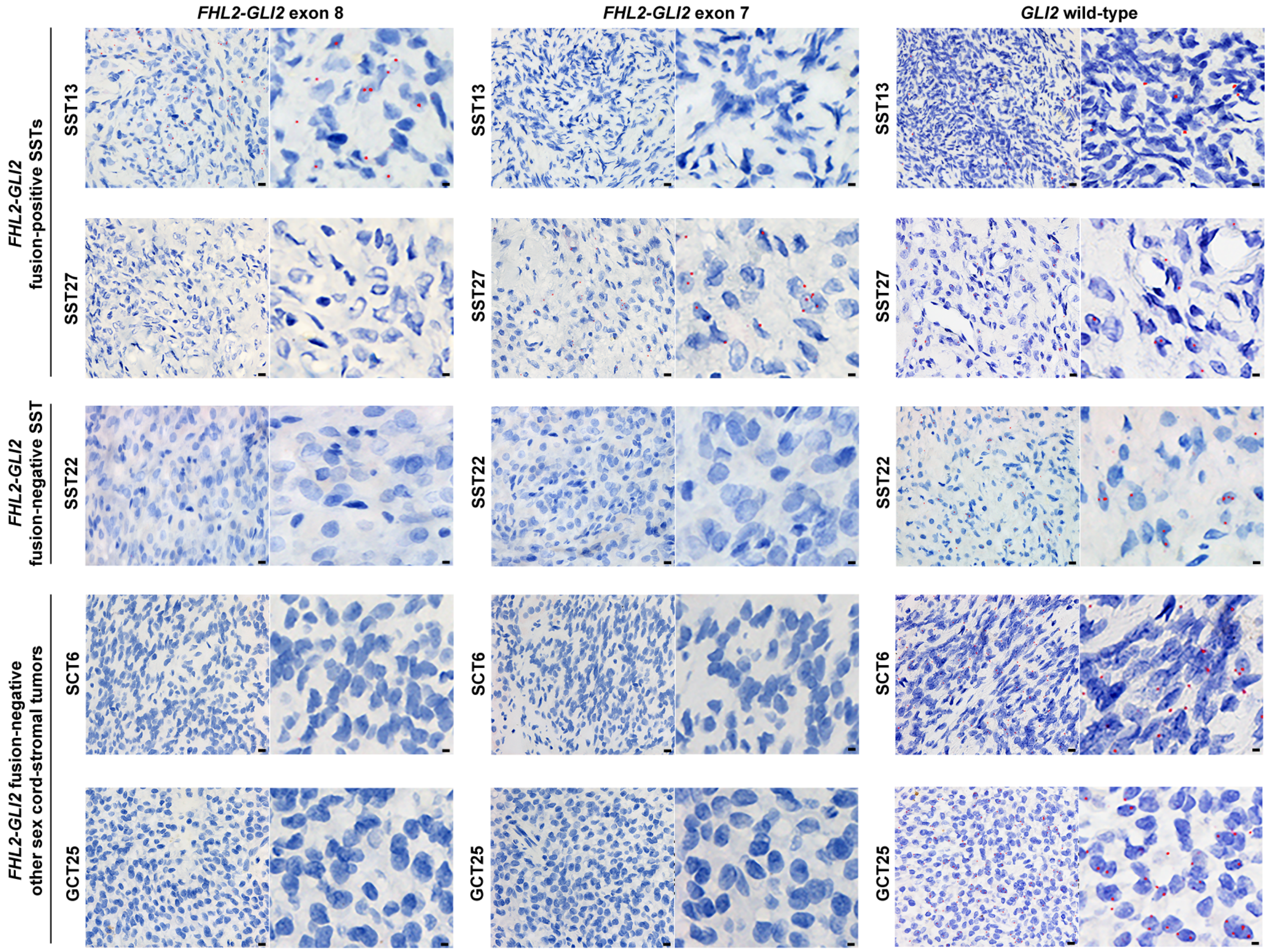

Supplementary Figure 6: RNA *in situ* hybridization (RNA-ISH) of sclerosing stromal tumors of the ovary and other sex cord-stromal tumors. RNA-ISH using custom probes (red dots) for *FHL2-GLI2* (*GLI2* exon 8; left), *FHL2-GLI2* (*GLI2* exon 7, middle) and wild-type *GLI2* (right). SSTs harboring *FHL2-GLI2* fusions (SST13, exon 8 *GLI2*; SST27, exon 7 *GLI2*; rows 1 and 2), SSTs lacking *FHL2-GLI2* fusions (middle row) and other sex cord-stromal tumors lacking *FHL2-GLI2* fusions (bottom two rows) were assessed. All cases expressed *GLI2* mRNA, whereas the expression of the chimeric *FHL2-GLI2* mRNA was restricted to SSTs harboring an *FHL2-GLI2* fusion. Scale bars 20 µm (low power magnification) and 40 µm (high power magnification).

Supplementary Figure 7

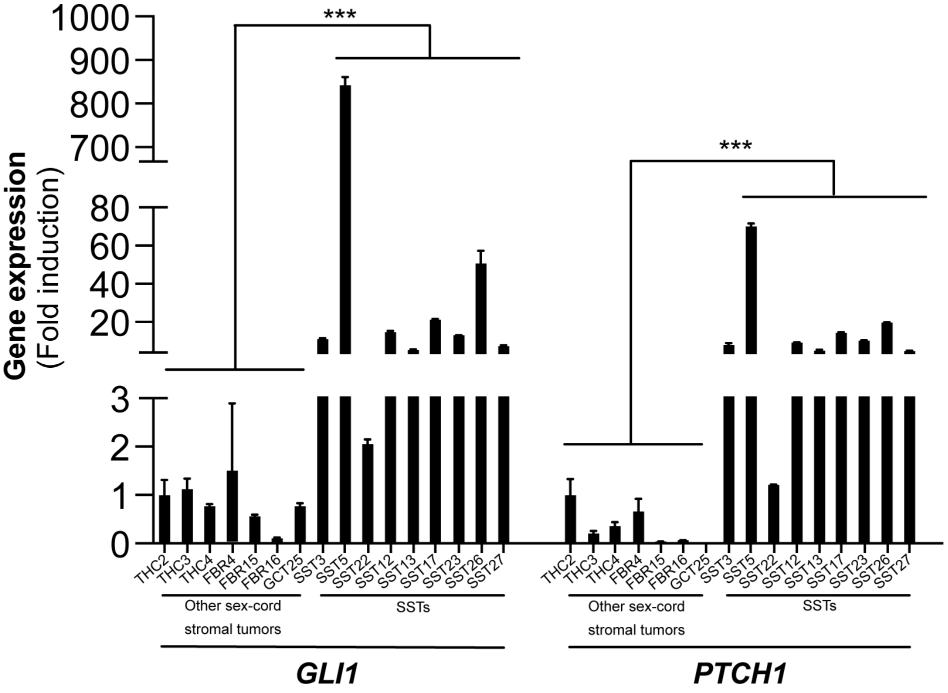

Supplementary Figure 7: Quantitative (q)PCR of *GLI1* and *PTCH1* in sclerosing stromal tumors of the ovary and other sex cord-stromal tumors. Bar plot depicting *GLI1* and *PTCH1* gene expression levels in sclerosing stromal tumors (SSTs) compared to other sex cord-stromal tumors. Error bars, s.d. of mean; *** P<0.001, Mann-Whitney U test, two-tailed.

Supplementary Figure 8

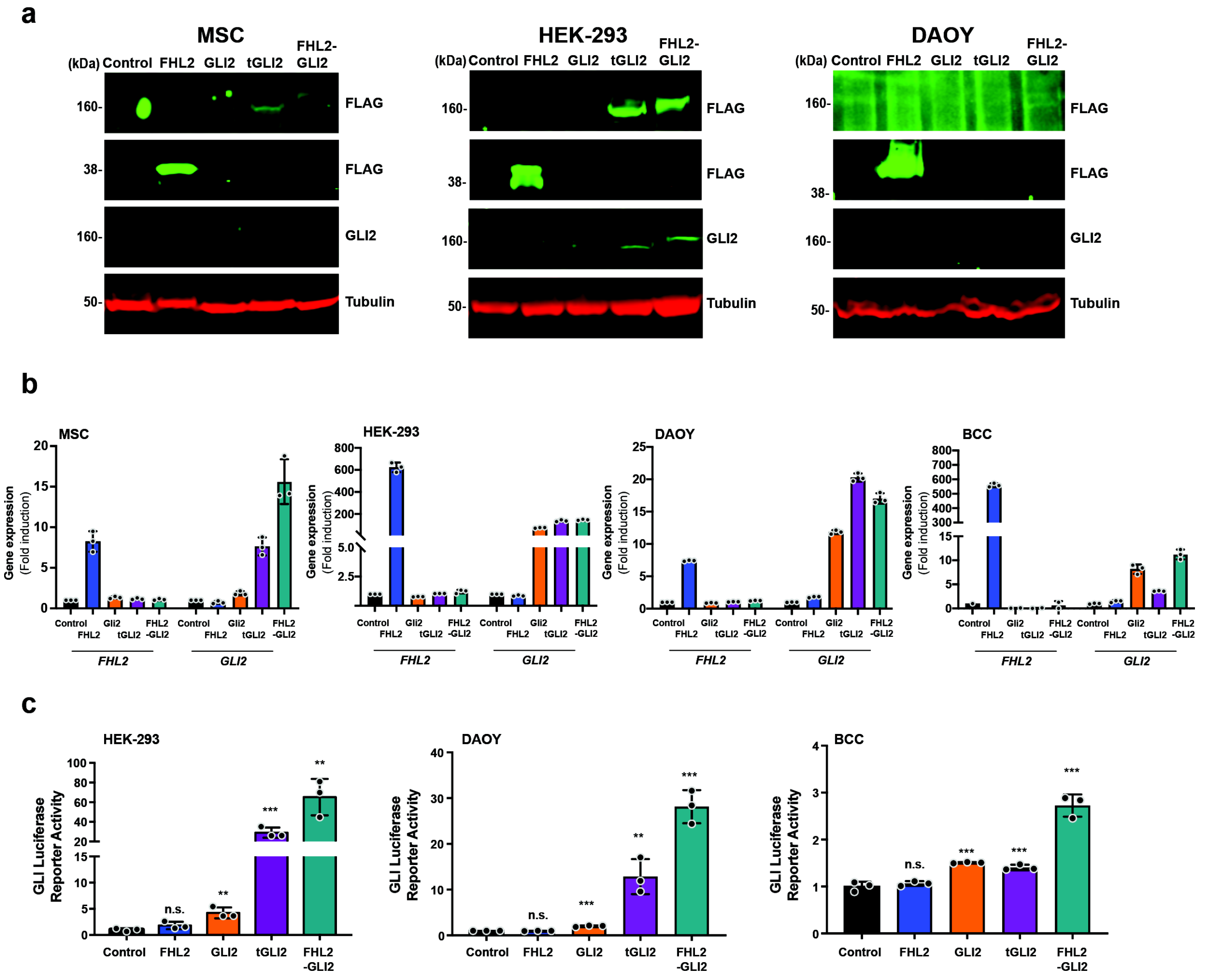

Supplementary Figure 8: Validation of cell models. (**a**) Representative western blot analysis of FLAG and GLI2 protein expression in MSC, HEK-293 and DAOY cells stably expressing empty vector (control), wild-type FHL2, wild-type GLI2, truncated GLI2 (tGLI2) and the FHL2-GLI2 fusion. (**b**) Quantitative assessment of *FHL2* and *GLI2* transcripts in MSC, HEK-293, DAOY and BCC cells stably expressing control, FHL2, GLI2, tGLI2 and FHL2-GLI2. (**c**) GLI luciferase reporter assay of HEK-293, DAOY and BCC cells stably expressing control, FHL2, GLI2, tGLI2 and FHL2-GLI2. In **b-c**, data are representative of at least three independent experiments. Error bars, s.d. of mean; n.s., not significant; ** $P < 0.01$, *** $P < 0.001$; two-tailed unpaired t-test.

Supplementary Figure 9

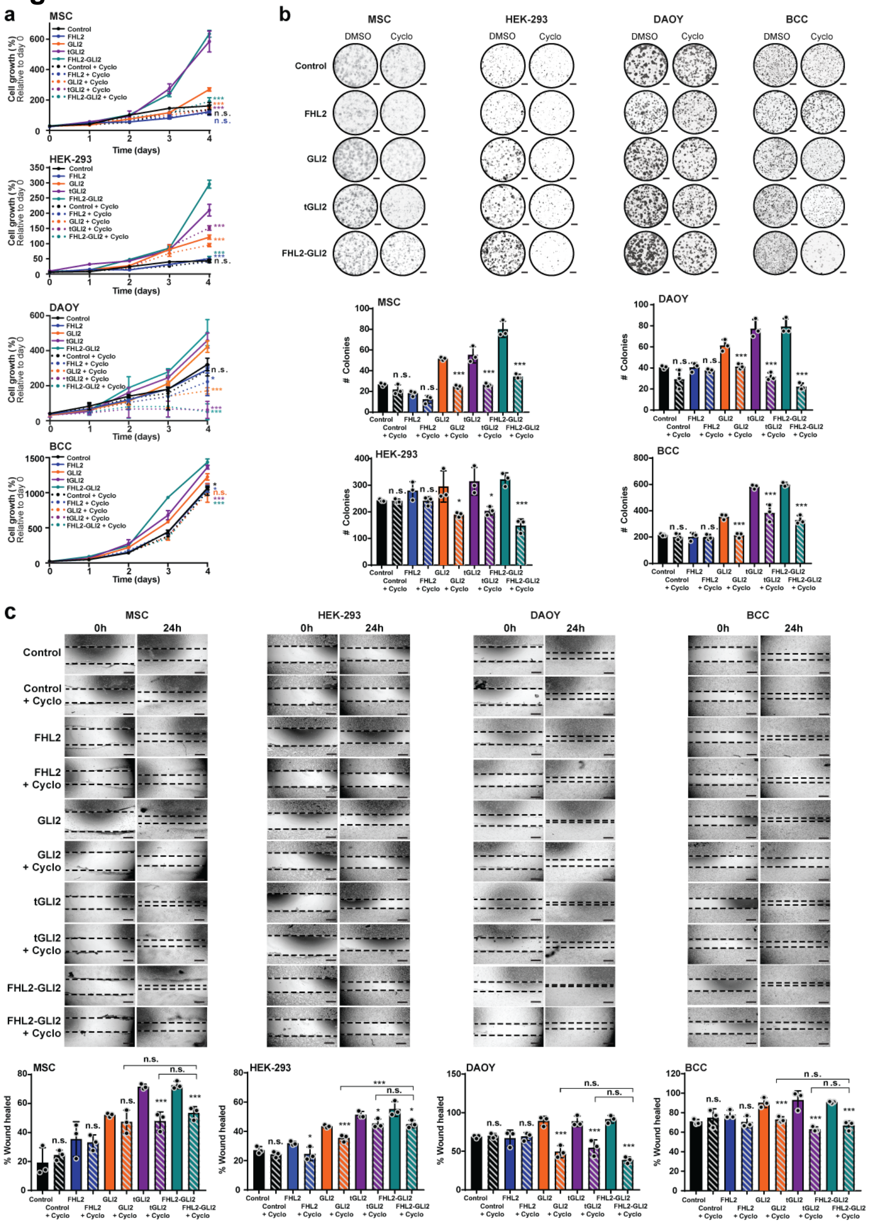

Supplementary Figure 9: Inhibition of the Sonic Hedgehog pathway using the SMO inhibitor Cyclopamine results in reversal of oncogenic properties in FHL2-GLI2 expressing cells *in vitro*.

a) Cell titer blue proliferation assay of immortalized mesenchymal stem cells (MSCs), HEK-293, medulloblastoma (DAOY) and human basal cell carcinoma (BCC) cells stably expressing empty vector (control), FHL2, GLI2, tGLI2 or FHL2-GLI2 treated with 10 µM Cyclopamine or vehicle control (DMSO). **b**) Representative images of colony formation assay of MSC, HEK-293, DAOY and BCC cells stably expressing control, FHL2, GLI2, tGLI2 or FHL2-GLI2 treated with 10 µM Cyclopamine or vehicle control (DMSO). Scale bars, 5 mm. Quantification of the number of colonies/well compared to control (bottom). **c**) Wound healing assay of MSC, HEK-293, DAOY and BCC cells stably expressing control, FHL2, GLI2, tGLI2 or FHL2-GLI2 treated with 10 µM Cyclopamine or vehicle control (DMSO). Cyclo, Cyclopamine. Scale bars, 500 µm. In **a-c**, data are representative of at least three independent experiments. Error bars, s.d. of mean; n.s., not significant; * $P < 0.05$, ** $P < 0.01$, *** $P < 0.001$; two-tailed unpaired t-test.

Supplementary Figure 10

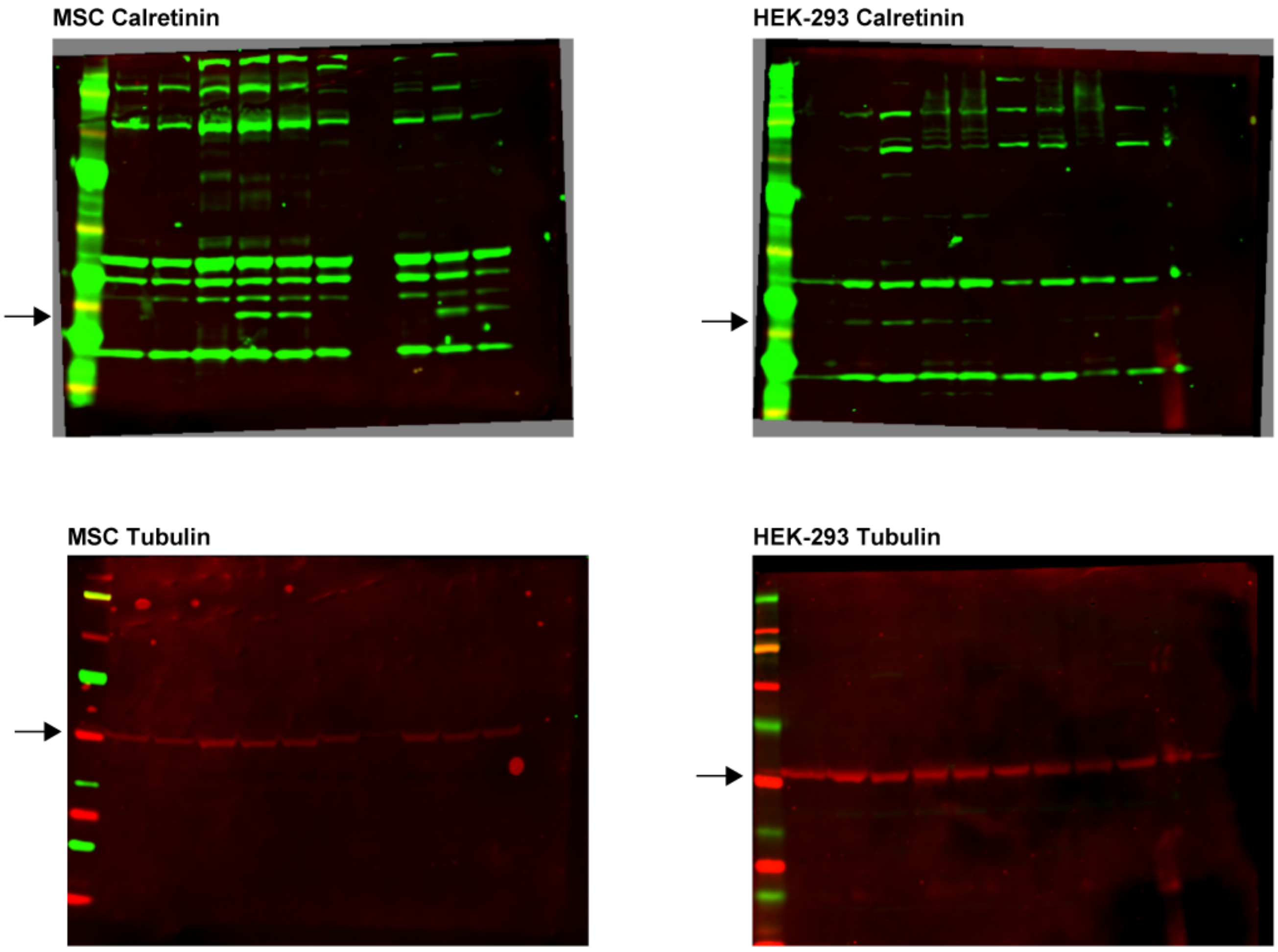

Supplementary Figure 10: Unprocessed images of western blots. Unprocessed images of scanned immunoblots shown in **Figure 3**. The arrows indicate the band of interest.

Supplementary Figure 11

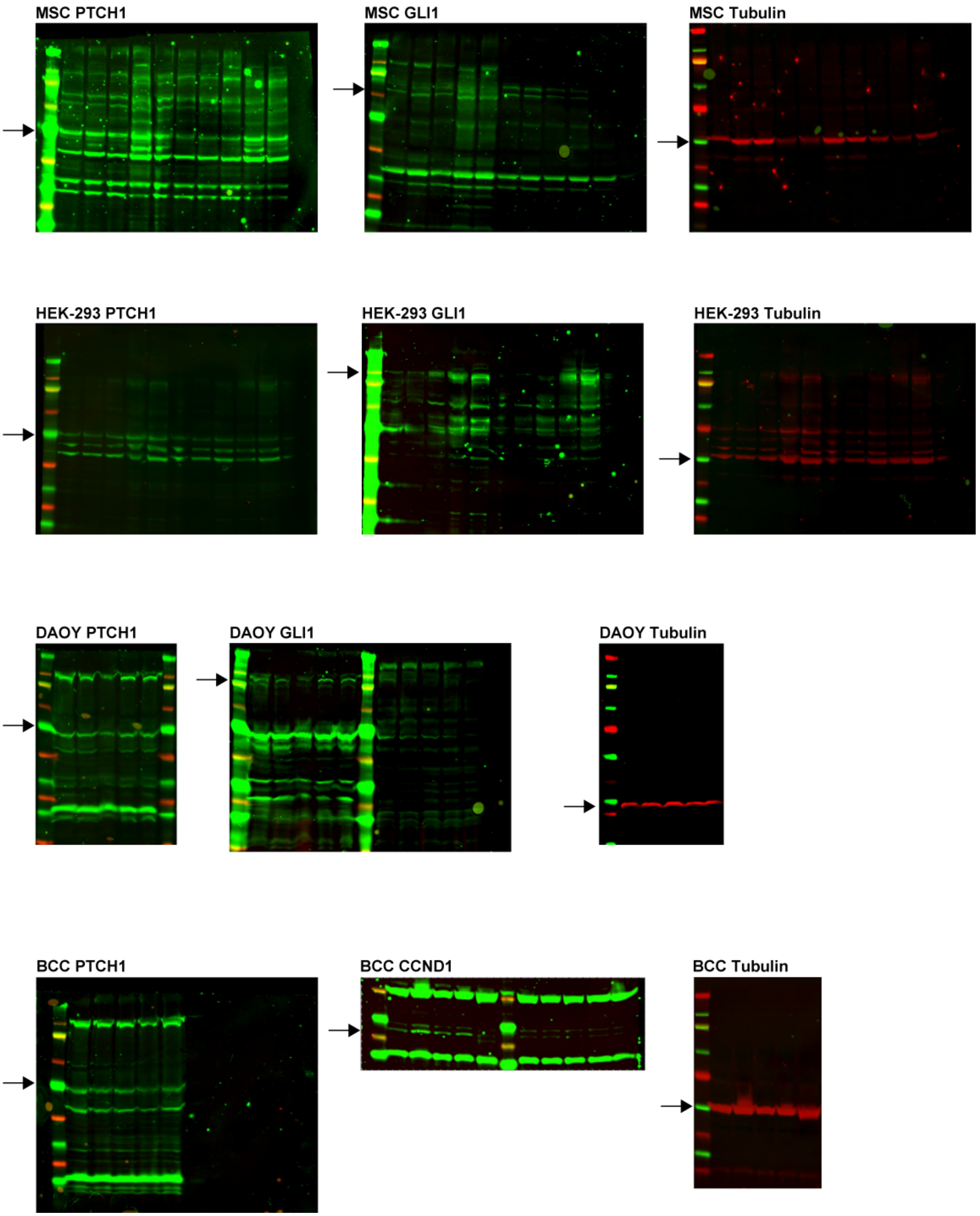

Supplementary Figure 11: Unprocessed images of western blots. Unprocessed images of scanned immunoblots shown in **Figure 4**. The arrows indicate the band of interest.

Supplementary Figure 12

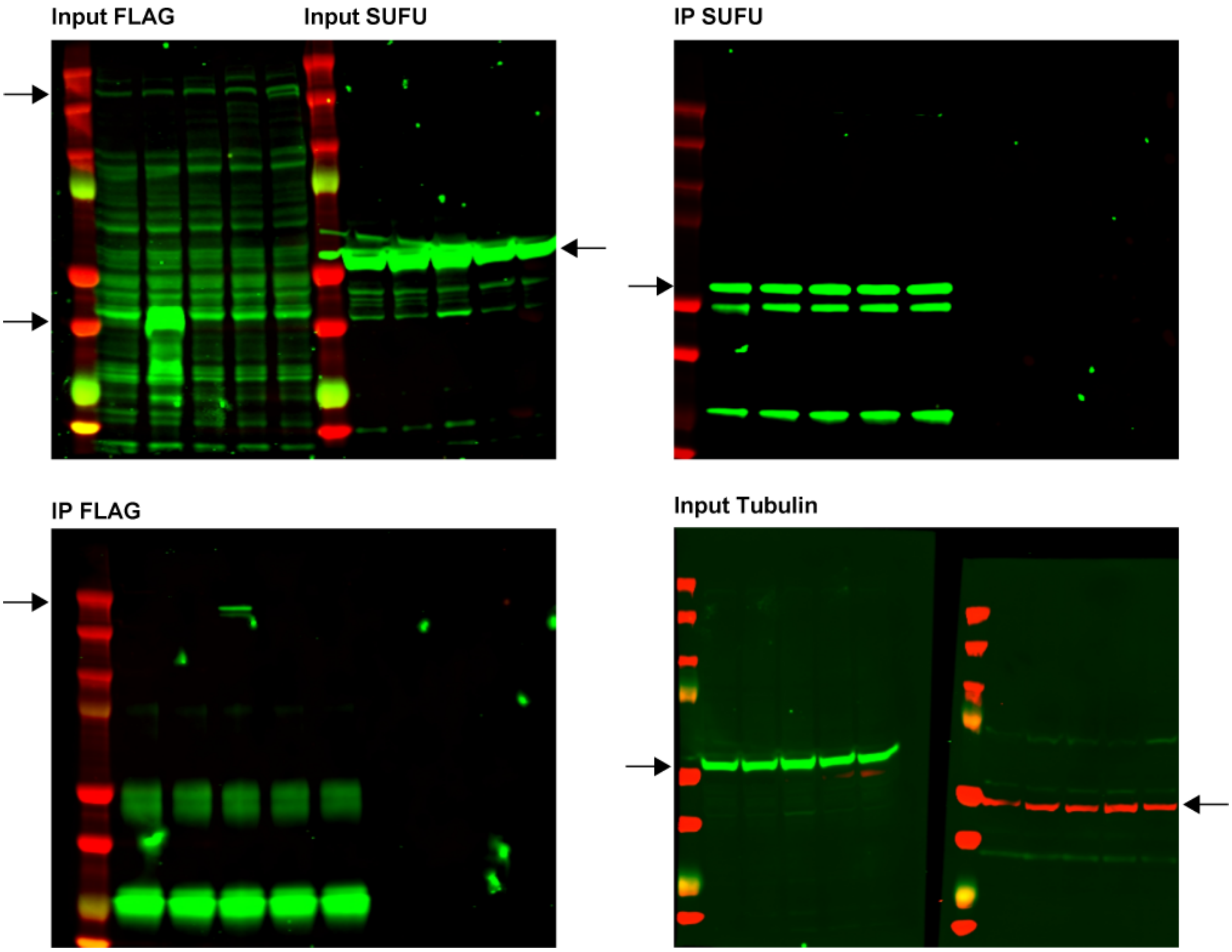

Supplementary Figure 12: Unprocessed images of western blots. Unprocessed images of scanned immunoblots shown in **Figure 5**. The arrows indicate the band of interest.

Supplementary Figure 13

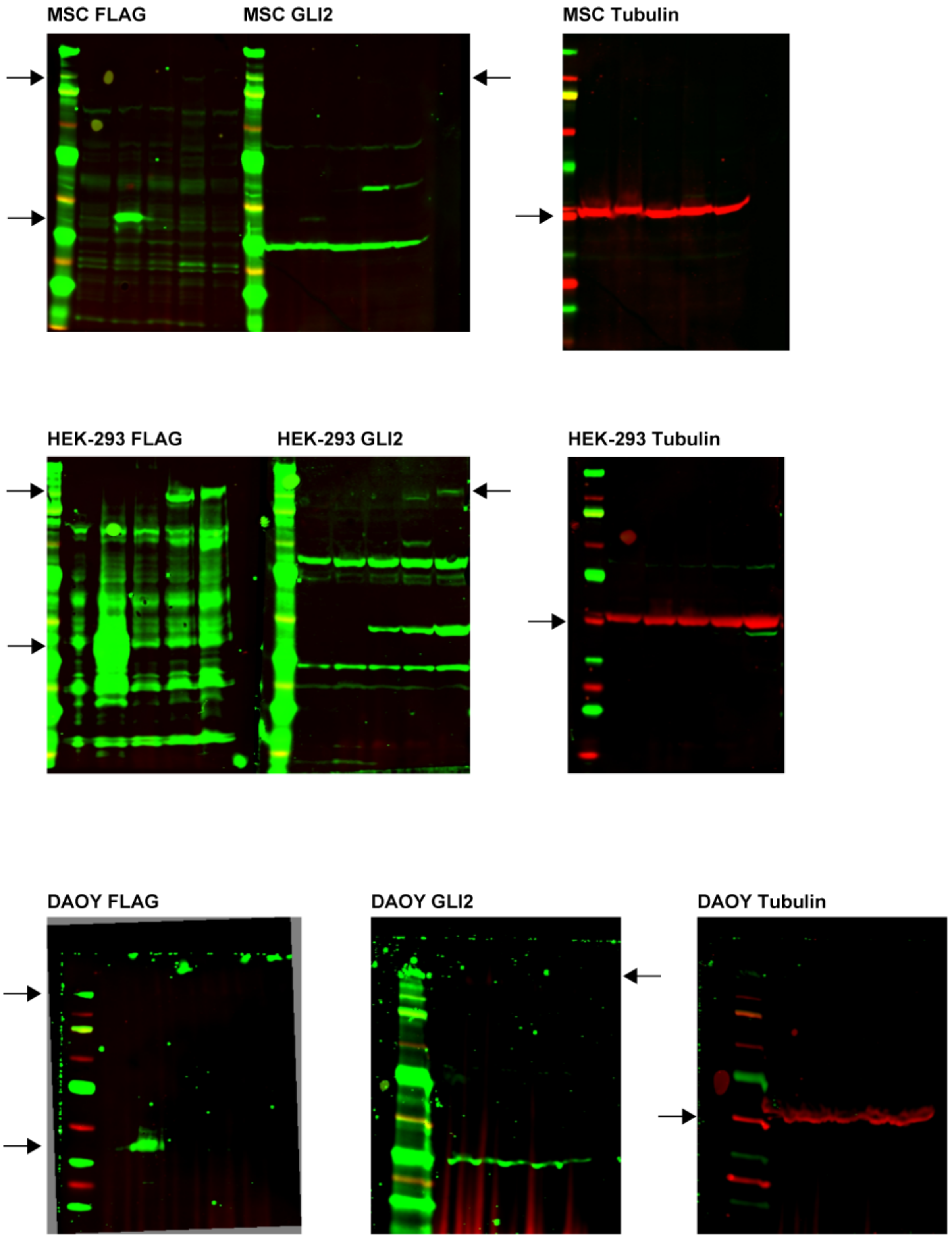

Supplementary Figure 13: Unprocessed images of western blots. Unprocessed images of scanned immunoblots shown in **Supplementary Figure 8**. The arrows indicate the band of interest.

Supplementary Table 1. Clinicopathologic characteristics of the sclerosing stromal tumors of the ovary included in this study, sequencing modalities employed and sequencing statistics.

	Case ID	Age at diagnosis (years)	Tumor site ovary	Surgery	Histopathological features						RNA-sequencing	DNA sequencing						*FHL-GLI2* fusion/ *GLI2* rearrangment status
					Lobulation	Staghorn vessels	Spindle cells	Luteinized cells	Cellularity	Mitoses		Sequencing	Total reads	Mean target coverage (x)	Target bases 2X	Target bases 50X	Target bases 100X	
Discovery	SST1	14	Right	Cystectomy	Vague	+	Minority	Majority	+++	1/10 HPF	Yes	MSK-IMPACT	47956698	698	0.992446	0.98273	0.968579	*GLI2* rearrangment
	SST2	41	Left	Hysterectomy, LSO	Vague	+	Less	More	+	-	Yes	WES	115163345	50	0.989455	0.43324	0.082295	*FHL2-GLI2*
	SST12	56	Right	Hysterectomy, BSO	Vague	+++	Minority	Majority	+++	<1/10 HPF	Yes	NP	NP	NP	NP	NP	NP	*DYNLL1-GLI2*
	SST22	20	Right	RSO	Vague	++	Minority	Majority	+++	1/10 HPF	Yes	NP	NP	NP	NP	NP	NP	Absent
	SST26	34	Left	LSO	Vague	+++	Minimal	Majority	+++	6/10 HPF	Yes	NP	NP	NP	NP	NP	NP	*FHL2-GLI2*
	SST27	29	Left	LSO	Minimal	++	Equal	Equal	+	<1/10 HPF	Yes	NP	NP	NP	NP	NP	NP	*FHL2-GLI2**
	SST28	19	Left	LSO	Vague	++	Equal	Equal	+ to ++	1/10 HPF	Yes	NP	NP	NP	NP	NP	NP	*FHL2-GLI2*
	SST29	31	Left	LSO	Vague to well formed	+ to ++	Less	More	+ to +++	1/10 HPF	Yes	NP	NP	NP	NP	NP	NP	*FHL2-GLI2*
Validation	SST3	20	Right	RSO	Vague	+	Minority	Majority	++	2/10 HPF	NP	NP	NP	NP	NP	NP	NP	Absent
	SST4	14	Right	Cystectomy	Vague	+	Equal	Equal	++	<1/10 HPF	NP	NP	NP	NP	NP	NP	NP	Absent
	SST5	25	Right	Bilateral cystectomy	N/A	N/A	N/A	N/A	N/A	N/A	NP	MSK-IMPACT	13855301	423	0.992223	0.974076	0.911303	Absent
	SST6	24	Right	Cystectomy	Vague	+	Equal	Equal	+	<1/10 HPF	NP	MSK-IMPACT	24010246	730	0.992598	0.984585	0.974161	*FHL2-GLI2*
	SST7	31	Right	Cystectomy	Mostly vague	+	Less	More	+++	<1/10 HPF	NP	MSK-IMPACT	28832851	856	0.99316	0.985776	0.97239	*FHL2-GLI2*
	SST8	23	Right	Cystectomy	Vague	+++	Equal	Equal	++	<1/10 HPF	NP	MSK-IMPACT	20004317	587	0.992908	0.980429	0.946028	*GLI2* rearrangment
	SST9	30	Left	LSO	Vague	++	Equal	Equal	++	<1/10 HPF	NP	WES	146410867	108	0.99731	0.863968	0.520906	*FHL2-GLI2*
	SST13	32	Right	BSO	Pronounced	+	Equal	Equal	+	<1/10 HPF	NP	NP	NP	NP	NP	NP	NP	*FHL2-GLI2*
	SST14	40	Right	RSO	Vague	+++	Equal	Equal	++	3/10 HPF	NP	NP	NP	NP	NP	NP	NP	*FHL2-GLI2*
	SST15	N/A	N/A	N/A	Vague	++	Equal	Equal	++	1/10 HPF	NP	NP	NP	NP	NP	NP	NP	Absent
	SST17	53	Right	Hysterectomy, BSO	Vague	+	Majority	Minority	+	<1/10 HPF	NP	WES	213327471	226	0.99773	0.85623	0.656396	*FHL2-GLI2*
	SST18	N/A	N/A	N/A	More pronounced	+++	Minority	Majority	+++	4/10 HPF	NP	NP	NP	NP	NP	NP	NP	*FHL2-GLI2*
	SST20	N/A	N/A	N/A	Vague	++	Less	More	++	3/10 HPF	NP	NP	NP	NP	NP	NP	NP	*GLI2* rearrangment
	SST21	N/A	N/A	N/A	Vague	++	Majority	Minority	+	<1/10 HPF	NP	NP	NP	NP	NP	NP	NP	*FHL2-GLI2*
	SST23	25	Right	RSO	Very vague	+	Equal	Equal	+	<1/10 HPF	NP	NP	NP	NP	NP	NP	NP	*FHL2-GLI2*
	SST24	40	Right	Hysterectomy, BSO	Vague	+++	Less	More	++	1/10 HPF	NP	NP	NP	NP	NP	NP	NP	*FHL2-GLI2*
	SST25	29	Left	LSO	N/A	N/A	N/A	N/A	N/A	N/A	NP	NP	NP	NP	NP	NP	NP	*FHL2-GLI2*
	SST30	31	Left	LSO	Vague	+++	Less	More	++	<1/10 HPF	NP	NP	NP	NP	NP	NP	NP	*FHL2-GLI2*

BSO, bilateral salpingo-oophorectomy, HPF, high power fields; LSO, left salpingo-oophorectomy; N/A, not available; NP, not performed; RSO, right salpingo-oophorectomy; WES, whole-exome sequencing; *, different breakpoint.

Supplementary Table 2. Non-synonymous somatic mutations identified in sclerosing stromal tumors of the ovary by whole-exome and MSK-IMPACT sequencing.

Sample ID	Gene symbol	Amino acid change	Chromosome	Genomic position	Reference allele	Alternate allele	Type of mutation	Depth at mutation (x)	Mutant allele fraction	Mutation hotspot	Cancer Cell Fraction (ABSOLUTE)	Clonal Status	Loss of heterozygosity (LOH)	Pathogenicity	Cancer driver gene (Bailey et al)	Cancer Gene Census
SST1	JAK3	p.Y425H	19	17950454	A	G	Missense_Mutation	347	44.4%	No	100%	Clonal	.	passenger	No	Yes
SST1	CIC	p.P663A	19	42794907	C	G	Missense_Mutation	289	45.3%	No	100%	Clonal	.	passenger	No	Yes
SST17	HCN4	p.P882S	15	73615790	G	A	Missense_Mutation	137	40.1%	No	100%	Clonal	.	passenger	No	No
SST17	PIP5K1C	p.Y221C	19	3653547	T	C	Missense_Mutation	249	34.9%	No	100%	Clonal	.	passenger	No	No
SST17	CD70	p.L53F	19	6590857	G	A	Missense_Mutation	451	34.8%	No	100%	Clonal	.	passenger	Yes	No
SST17	GRM7	p.T182M	3	7188164	C	T	Missense_Mutation	72	6.9%	No	39%	Subclonal	.	likely_pathogenic	No	No
SST17	WDR6	p.L624Q	3	49050748	T	A	Missense_Mutation	300	7.3%	No	42%	Subclonal	.	passenger	No	No
SST17	CMYA5	p.R3777Q	5	79057703	G	A	Missense_Mutation	110	5.5%	No	31%	Subclonal	.	passenger	No	No
SST17	CALN1	p.R88W	7	71571262	G	A	Missense_Mutation	218	3.7%	No	21%	Subclonal	.	passenger	No	No
SST17	KIF24	p.P349Q	9	34290253	G	T	Missense_Mutation	70	22.9%	No	100%	Clonal	.	passenger	No	No
SST17	ST8SIA4	p.R203*	5	100191997	G	A	Nonsense_Mutation	66	7.6%	No	43%	Subclonal	.	passenger	No	No
SST2	HGF	p.P676L	7	81332057	G	A	Missense_Mutation	94	5.3%	No	33%	Subclonal	.	likely_pathogenic	Yes	No
SST2	FGFR1	p.S140L	8	38287238	G	A	Missense_Mutation	80	17.5%	No	100%	Clonal	Yes	passenger	Yes	Yes
SST2	ATR	p.R1183*	3	142259780	G	A	Nonsense_Mutation	75	6.7%	No	41%	Subclonal	.	likely_pathogenic	Yes	Yes
SST8	FGFR3	p.Q29H	4	1795748	G	C	Missense_Mutation	560	43.8%	No	89%	Subclonal	.	passenger	Yes	Yes
SST8	HIST1H1C	p.A123V	6	26056289	G	A	Missense_Mutation	155	44.5%	No	91%	Clonal	.	passenger	Yes	No
SST8	ARID1B	p.A1935V	6	157528079	C	T	Missense_Mutation	149	38.3%	No	78%	Subclonal	.	passenger	No	Yes
SST9	CKAP5	p.G255D	11	46831050	C	T	Missense_Mutation	172	31.4%	No	84%	Subclonal	.	passenger	No	No
SST9	STARD9	p.R51G	15	42877752	C	G	Missense_Mutation	117	30.8%	No	82%	Subclonal	.	passenger	No	No
SST9	PLCL1	p.Q368R	2	198949344	A	G	Missense_Mutation	160	33.8%	No	90%	Clonal	.	passenger	No	No
SST9	IQCA1	p.A327P	2	237349715	C	G	Missense_Mutation	115	32.2%	No	86%	Clonal	.	passenger	No	No
SST9	SLC4A7	p.R82Q	3	27490159	C	T	Missense_Mutation	104	26.9%	No	72%	Subclonal	.	likely_pathogenic	No	No
SST9	TCAIM	p.R217H	3	44434424	G	A	Missense_Mutation	74	25.7%	No	69%	Subclonal	.	passenger	No	No
SST9	FAM160A1	p.L397F	4	152559873	A	T	Missense_Mutation	91	28.6%	No	76%	Subclonal	Yes	passenger	No	No
SST9	PDZD2	p.I608V	5	32053911	A	G	Missense_Mutation	189	31.2%	No	83%	Subclonal	.	passenger	No	No
SST9	DNAH11	p.M924I	7	21639509	G	A	Missense_Mutation	180	38.3%	No	100%	Clonal	.	passenger	No	No
SST9	RP1L1	p.G1335V	8	10467604	C	A	Missense_Mutation	470	32.1%	No	86%	Subclonal	Yes	passenger	No	No

Supplementary Table 3. Fusion candidate genes and/or readthroughs identified by RNA-sequencing analysis of sclerosing stromal tumors of the ovary.

Case ID	Fusion Caller	5' Gene	3' Gene	5' Mapping	3' Mapping	Fusion Type	Crossing Reads	Encompassing Reads	In Frame	Driver Probability (Oncofuse)	RT-PCR primer sets
SST2	BOWTIE-Integrate, BWA-Integrate, STAR-Integrate, FusionCatcher, Defuse	*FHL2*	*GLI2*	2:105984027-	2:121732551+	Intra_Chromosomal	10	26	Yes	0.999938302	F1: TGCCCTGCTATGAGAAACAA<br>R1: TGCTTACAGTCATCCCTGTCC<br>F2: CATCCCCAAAGACAATCAGAA<br>R2: CCTCAGCCTCCTGCTTACAG
SST26	BOWTIE-Integrate, BWA-Integrate, STAR-Integrate, FusionCatcher, Defuse	*FHL2*	*GLI2*	2:105984027-	2:121732551+	Intra_Chromosomal	4	47	Yes	0.999938302	F1: TGCCCTGCTATGAGAAACAA<br>R1: TGCTTACAGTCATCCCTGTCC<br>F2: CATCCCCAAAGACAATCAGAA<br>R2: CCTCAGCCTCCTGCTTACAG
SST28	BOWTIE-Integrate, BWA-Integrate, STAR-Integrate, FusionCatcher, Defuse	*FHL2*	*GLI2*	2:105984027-	2:121732551+	Intra_Chromosomal	14	100	Yes	0.999938302	F1: TGCCCTGCTATGAGAAACAA<br>R1: TGCTTACAGTCATCCCTGTCC<br>F2: CATCCCCAAAGACAATCAGAA<br>R2: CCTCAGCCTCCTGCTTACAG
SST29	BOWTIE-Integrate, BWA-Integrate, STAR-Integrate, FusionCatcher, Defuse	*FHL2*	*GLI2*	2:105984027-	2:121732551+	Intra_Chromosomal	28	55	Yes	0.999938302	F1: TGCCCTGCTATGAGAAACAA<br>R1: TGCTTACAGTCATCCCTGTCC<br>F2: CATCCCCAAAGACAATCAGAA<br>R2: CCTCAGCCTCCTGCTTACAG
SST12	BOWTIE-Integrate, BWA-Integrate, STAR-Integrate, FusionCatcher, Defuse	*DYNLL1*	*GLI2*	12:120934356+	2:121732551+	Inter_Chromosomal	11	22	No	0.762626377	F1: AAGAGATGCAACAGGACTCG<br>R1: CAGTCTTCCCAGTGGCAGTT<br>F2: TGTCGGAAGAGATGCAACAG<br>R2: ATGACCACCTCAGCCTCCT
SST27	BOWTIE-Integrate, BWA-Integrate, STAR-Integrate, FusionCatcher, Defuse	*FHL2*	*GLI2*	2:106002818:-	2:121729517:+	Intra_Chromosomal	9	23	No	0.999938302	F1: GGTGTGCTTTGAGACCCTGT<br>R1: CAGGCTCGGTCTTGACCTT<br>F2: CTACTGCGTGGTGTGCTTTG<br>R2: CTCGGTCTTGACCTTGCTG

Supplementary Table 4. *FHL2-GLI2* fusion gene assessment by RT-PCR, fluorescence *in situ* hybridization and RNA *in situ* hybridization in sclerosing stromal tumors of the ovary and in other ovarian sex cord-stromal tumors.

	Sample ID	Tumor type	RT-PCR	*FHL2-GLI2* fusion by RT-PCR	*GLI2* rearrangements by RT-PCR	FISH	*FHL2-GLI2* fusion by FISH	*GLI2* rearrangements by FISH	RNA-ISH	*FHL2-GLI2* mRNA expression by RNA-ISH
**Discovery**	SST1	Sclerosing stromal tumor	Yes	Absent	NP	Yes	Absent	Present	No	NP
	SST2	Sclerosing stromal tumor	Yes	Present	NP	Yes	Present	Present	No	NP
	SST12	Sclerosing stromal tumor	Yes	Absent	Present	No	NP	NP	No	NP
	SST22	Sclerosing stromal tumor	Yes	Absent	NP	Yes	Absent	Absent	Yes	Absent
	SST26	Sclerosing stromal tumor	Yes	Present	NP	No	NP	NP	No	NP
	SST27	Sclerosing stromal tumor	Yes	Present*	NP	No	NP	NP	Yes	Present*
	SST28	Sclerosing stromal tumor	Yes	Present	NP	No	NP	NP	No	NP
	SST29	Sclerosing stromal tumor	Yes	Present	NP	No	NP	NP	No	NP
**Validation**	SST3	Sclerosing stromal tumor	Yes	Absent	NP	Yes	Absent	Absent	No	NP
	SST4	Sclerosing stromal tumor	Yes	Absent	NP	No	NP	NP	No	NP
	SST5	Sclerosing stromal tumor	Yes	Absent	NP	Yes	Absent	Absent	No	NP
	SST6	Sclerosing stromal tumor	Yes	Present	NP	Yes	Present	Present	No	NP
	SST7	Sclerosing stromal tumor	Yes	Present	NP	Yes	Present	Present	No	NP
	SST8	Sclerosing stromal tumor	Yes	Absent	NP	Yes	Absent	Present	No	NP
	SST9	Sclerosing stromal tumor	Yes	Present	NP	Yes	Present	Present	No	NP
	SST13	Sclerosing stromal tumor	Yes	Present	NP	Yes	Present	Present	Yes	Present
	SST14	Sclerosing stromal tumor	Yes	Present	NP	No	NP	NP	No	NP
	SST15	Sclerosing stromal tumor	Yes	Absent	NP	Yes	Absent	NP	No	NP
	SST17	Sclerosing stromal tumor	Yes	Present	NP	Yes	Present	Present	Yes	Present
	SST18	Sclerosing stromal tumor	Yes	Present	NP	No	NP	NP	No	NP
	SST20	Sclerosing stromal tumor	Yes	Absent	NP	Yes	Absent	Present	No	NP
	SST21	Sclerosing stromal tumor	Yes	Present	NP	No	NP	NP	No	NP
	SST23	Sclerosing stromal tumor	Yes	Present	NP	No	NP	NP	Yes	Present
	SST24	Sclerosing stromal tumor	Yes	Present	NP	No	NP	NP	No	NP
	SST25	Sclerosing stromal tumor	Yes	Present	NP	No	NP	NP	No	NP
	SST30	Sclerosing stromal tumor	Yes	Present	NP	No	NP	NP	No	NP
**Other ovarian sex cord-stromal tumors**	GCT1	Granulosa cell tumor	Yes	Absent	NP	No	NP	NP	No	NP
	GCT2	Granulosa cell tumor	Yes	Absent	NP	No	NP	NP	No	NP
	GCT3	Granulosa cell tumor	Yes	Absent	NP	No	NP	NP	No	NP
	GCT4	Granulosa cell tumor	Yes	Absent	NP	No	NP	NP	No	NP
	GCT5	Granulosa cell tumor	Yes	Absent	NP	No	NP	NP	No	NP
	GCT6	Granulosa cell tumor	Yes	Absent	NP	No	NP	NP	No	NP
	GCT7	Granulosa cell tumor	Yes	Absent	NP	No	NP	NP	No	NP
	GCT8	Granulosa cell tumor	Yes	Absent	NP	No	NP	NP	No	NP
	GCT9	Granulosa cell tumor	Yes	Absent	NP	No	NP	NP	No	NP
	GCT10	Granulosa cell tumor	Yes	Absent	NP	No	NP	NP	No	NP
	GCT11	Granulosa cell tumor	Yes	Absent	NP	No	NP	NP	No	NP
	GCT12	Granulosa cell tumor	Yes	Absent	NP	No	NP	NP	No	NP
	GCT13	Granulosa cell tumor	Yes	Absent	NP	No	NP	NP	No	NP
	GCT14	Granulosa cell tumor	Yes	Absent	NP	No	NP	NP	No	NP
	GCT15	Granulosa cell tumor	Yes	Absent	NP	No	NP	NP	No	NP
	GCT16	Granulosa cell tumor	Yes	Absent	NP	No	NP	NP	No	NP
	GCT17	Granulosa cell tumor	Yes	Absent	NP	No	NP	NP	No	NP
	GCT18	Granulosa cell tumor	Yes	Absent	NP	No	NP	NP	No	NP
	GCT19	Granulosa cell tumor	Yes	Absent	NP	No	NP	NP	No	NP
	GCT20	Granulosa cell tumor	Yes	Absent	NP	No	NP	NP	No	NP
	GCT21	Granulosa cell tumor	Yes	Absent	NP	No	NP	NP	No	NP
	GCT22	Granulosa cell tumor	Yes	Absent	NP	No	NP	NP	No	NP
	GCT23	Granulosa cell tumor	Yes	Absent	NP	No	NP	NP	No	NP
	GCT24	Granulosa cell tumor	Yes	Absent	NP	Yes	Absent	Absent	No	NP
	GCT25	Granulosa cell tumor	Yes	Absent	NP	Yes	Absent	Absent	No	NP
	GCT26	Granulosa cell tumor	Yes	Absent	NP	Yes	Absent	Absent	Yes	Absent
	GCT27	Granulosa cell tumor	Yes	Absent	NP	Yes	Absent	Absent	No	NP
	GCT28	Granulosa cell tumor	Yes	Absent	NP	Yes	Absent	Absent	No	NP
	FBR3	Fibroma	Yes	Absent	NP	No	NP	NP	No	NP
	FBR4	Fibroma	Yes	Absent	NP	No	NP	NP	Yes	Absent
	FBR6	Fibroma	Yes	Absent	NP	No	NP	NP	No	NP
	FBR7	Fibroma	Yes	Absent	NP	No	NP	NP	No	NP
	FBR10	Fibroma	Yes	Absent	NP	No	NP	NP	No	NP
	FBR12	Fibroma	Yes	Absent	NP	No	NP	NP	No	NP
	FBR14	Fibroma	Yes	Absent	NP	No	NP	NP	No	NP
	FBR15	Fibroma	Yes	Absent	NP	Yes	Absent	Absent	No	NP
	FBR16	Fibroma	Yes	Absent	NP	No	NP	NP	No	NP
	FBT3	Fibrothecoma	Yes	Absent	NP	No	NP	NP	No	NP
	THC1	Thecoma	Yes	Absent	NP	No	NP	NP	No	NP
	THC2	Thecoma	Yes	Absent	NP	No	NP	NP	Yes	Absent
	THC3	Thecoma	Yes	Absent	NP	Yes	Absent	Absent	No	NP
	THC4	Thecoma	Yes	Absent	NP	No	NP	NP	No	NP
	SLCT1	Sertoli-Leydig cell tumor	Yes	Absent	NP	No	NP	NP	No	NP
	SLCT2	Sertoli-Leydig cell tumor	Yes	Absent	NP	No	NP	NP	No	NP
	SLCT3	Sertoli-Leydig cell tumor	Yes	Absent	NP	No	NP	NP	No	NP
	SLCT4	Sertoli-Leydig cell tumor	Yes	Absent	NP	No	NP	NP	No	NP
	SLCT5	Sertoli-Leydig cell tumor	Yes	Absent	NP	Yes	Absent	Absent	No	NP
	SLCT6	Sertoli-Leydig cell tumor	Yes	Absent	NP	Yes	Absent	Absent	Yes	Absent

*Distinct *FHL2-GLI2* breakpoint; FISH, fluorescence *in situ* hybridization; RNA-ISH, RNA *in situ* hybridization; NP, not performed.

Supplementary Table 5. Cloning and sequencing primers.

Primer pairing	5' - 3' sequence
**WT FHL2 PCR primer pairs**	
CD513B-Flag-F	TGACCTCCATAGAAGATTCTAGAGCTAGCGgccaccatgactgagcgctttgactgccac
FHL2-CD513B-R	CGGAGCGATCGCAGATCCTTCGCGGCCGCGGATCCTAACCTCCACCTCCACCAGC<br>GTAATCTGGAACATCGTATGGGTAACCTCCACCGCCCCCTTTATCGTCATCGTCTTT<br>GTAGTCATCAAGAATACCTCCACCGCCACCAGAAATGCCAGGGATGTCTTTCCCACA<br>GTCGGGGCAC
**WT GLI2 PCR primer pairs**	
CD513B-Flag-F	TGACCTCCATAGAAGATTCTAGAGCTAGCGgccaccatggagacgtctgcctcagccactg
Gli2-CD513B-R	CGGAGCGATCGCAGATCCTTCGCGGCCGCGGATCCTAACCTCCACCTCCACCAGC<br>GTAATCTGGAACATCGTATGGGTAACCTCCACCGCCCCCTTTATCGTCATCGTCTTT<br>GTAGTCATCAAGAATACCTCCACCGCCACCAGAAATGCCAGGGGTCATCATGTTCA<br>GGAACTTGCTC
**Truncated GLI2 PCR primer pairs**	
CD513B-Flag-F	TGACCTCCATAGAAGATTCTAGAGCTAGCGgccaccatggagcagctggctgacctcaaggaag
Gli2-CD513B-R	CGGAGCGATCGCAGATCCTTCGCGGCCGCGGATCCTAACCTCCACCTCCACCAGC<br>GTAATCTGGAACATCGTATGGGTAACCTCCACCGCCCCCTTTATCGTCATCGTCTTT<br>GTAGTCATCAAGAATACCTCCACCGCCACCAGAAATGCCAGGGGTCATCATGTTCA<br>GGAACTTGCTCCCAGAAATGCCAGGGGTCATCATGTTCAGGAACTTGCTC
**FHL2-GLI2 fusion PCR primer pairs**	
CD513B-Flag-F	TGACCTCCATAGAAGATTCTAGAGCTAGCGgccaccatgactgagcgctttgactgccac
Gli2-CD513B-R	CGGAGCGATCGCAGATCCTTCGCGGCCGCGGATCCTAACCTCCACCTCCACCAGC<br>GTAATCTGGAACATCGTATGGGTAACCTCCACCGCCCCCTTTATCGTCATCGTCTTT<br>GTAGTCATCAAGAATACCTCCACCGCCACCAGAAATGCCAGGGGTCATCATGTTCA<br>GGAACTTGCTC
**Cloning primers**	
CD5131B-Flag-F	CTGTGACCGGCGCCTACTCTAGAGCTAGCGAATTCGCCACCATGGACTACAAAGACG
FHL2-CD513B-R	TGGCAACTAGAAGGCACAGTCGGCGGCCGCGGATCCCTAGATGTCTTTCCCACAGT
Gli2-CD513B-R	TGGCAACTAGAAGGCACAGTCGGCGGCCGCGGATCCCTAGGTCATCATGTTCAGGA
**Sequencing primers**	
CMV-f	CGCAAATGGGCGGTAGGCGTG

Supplementary Table 6. Chromatin immunoprecipitation (ChIP) quantitative (q)PCR primers.

**ChIP-qPCR primers**	
GLI1_promoter_1_F	CCACTGCCAGCCTGTGTATC
GLI1_promoter_1_R	AGTGCTGATTGTTTCGGGGT
GLI1_promoter_2_F	CGGGGGATATGTAAGGGTCG
GLI1_promoter_2_R	GGACACCTTAGGTTGCTGGT
GLI1_gene body_F	CCATATCCGTCTCCGCTGTC
GLI1_gene body_R	GAGGTGAGATGGACAGTGCC
PTCH1_promoter_1_F	CAGCGCGGACTCACAATTA
PTCH1_promoter_1_R	TGATAGCACGTTCCTCGAGTT
PTCH1_promoter_2_F	CTCTGAGAGCGCCAACCTTC
PTCH1_promoter_2_R	GTCTACCGCGAGGCAAATG
PTCH1_gene body_F	CACGTGTCCCCTGAGGTTTT
PTCH1_gene body_R	AGCAGAAGATTTGCCAACGC
MYOD1_promoter_F	AAAGTTCCGGCCACTCTCTG
MYOD1_promoter_R	TAGAAGTCGTCCGTTGTGGC
