# Supplementary material for: Identification of recurrent FHL2-GLI2 oncogenic fusion in sclerosing stromal tumors of the ovary
Source: Nat Commun. 2020 Jan 2;11:44. doi: 10.1038/s41467-019-13806-x (PMC6940380; doi:10.1038/s41467-019-13806-x)
Supplement: Supplementary file 2 — Reporting Summary [file 41467_2019_13806_MOESM2_ESM.pdf]

nature research

Corresponding author(s): Britta Weigelt

Last updated by author(s): Nov 13, 2019

Reporting Summary

Nature Research wishes to improve the reproducibility of the work that we publish. This form provides structure for consistency and transparency in reporting. For further information on Nature Research policies, see Authors & Referees and the Editorial Policy Checklist.

Statistics

For all statistical analyses, confirm that the following items are present in the figure legend, table legend, main text, or Methods section.

n/a	Confirmed	
☐	☒	The exact sample size (*n*) for each experimental group/condition, given as a discrete number and unit of measurement
☐	☒	A statement on whether measurements were taken from distinct samples or whether the same sample was measured repeatedly
☐	☒	The statistical test(s) used AND whether they are one- or two-sided<br>*Only common tests should be described solely by name; describe more complex techniques in the Methods section.*
☒	☐	A description of all covariates tested
☐	☒	A description of any assumptions or corrections, such as tests of normality and adjustment for multiple comparisons
☐	☒	A full description of the statistical parameters including central tendency (e.g. means) or other basic estimates (e.g. regression coefficient) AND variation (e.g. standard deviation) or associated estimates of uncertainty (e.g. confidence intervals)
☒	☐	For null hypothesis testing, the test statistic (e.g. *F*, *t*, *r*) with confidence intervals, effect sizes, degrees of freedom and *P* value noted<br>*Give P values as exact values whenever suitable.*
☒	☐	For Bayesian analysis, information on the choice of priors and Markov chain Monte Carlo settings
☒	☐	For hierarchical and complex designs, identification of the appropriate level for tests and full reporting of outcomes
☒	☐	Estimates of effect sizes (e.g. Cohen's *d*, Pearson's *r*), indicating how they were calculated

Our web collection on statistics for biologists contains articles on many of the points above.

Software and code

Policy information about availability of computer code

Data collection	Not applicable
Data analysis	No customized algorithms or softwares were utilized in this manuscript.

For manuscripts utilizing custom algorithms or software that are central to the research but not yet described in published literature, software must be made available to editors/reviewers. We strongly encourage code deposition in a community repository (e.g. GitHub). See the Nature Research guidelines for submitting code & software for further information.

Data

Policy information about availability of data

All manuscripts must include a data availability statement. This statement should provide the following information, where applicable:

- Accession codes, unique identifiers, or web links for publicly available datasets
- A list of figures that have associated raw data
- A description of any restrictions on data availability

RNA-Sequencing data have been deposited in Sequence Read Archive (SRA) with accession code PRJNA540984 (https://www.ncbi.nlm.nih.gov/sra/PRJNA540984). WES and targeted sequencing data are available via cBioPortal for Cancer Genomics (www.cbioportal.org). The remaining data are available in the Article or Supplementary Material.

October 2018

Field-specific reporting

Please select the one below that is the best fit for your research. If you are not sure, read the appropriate sections before making your selection.

☒ Life sciences ☐ Behavioural & social sciences ☐ Ecological, evolutionary & environmental sciences

For a reference copy of the document with all sections, see nature.com/documents/nr-reporting-summary-flat.pdf

Life sciences study design

All studies must disclose on these points even when the disclosure is negative.

Sample size	Assuming that sclerosing stromal tumors of the ovary would be driven by a recurrent genetic alteration present in ≥70% of cases, akin to other rare ovarian tumor types, based on a binomial distribution, sequencing of eight cases would be sufficient to identify a recurrent genetic alteration with >90% statistical power. This is stated in the Results section of the manuscript.
Data exclusions	No data were excluded.
Replication	For all in vitro experiments at least three independent replicates were performed. All replicates were successful, and the mean and standard deviation reported.
Randomization	Randomization was not performed.
Blinding	Blinding was not performed.

Reporting for specific materials, systems and methods

We require information from authors about some types of materials, experimental systems and methods used in many studies. Here, indicate whether each material, system or method listed is relevant to your study. If you are not sure if a list item applies to your research, read the appropriate section before selecting a response.

Materials & experimental systems

n/a	Involved in the study	
☐	☒	Antibodies
☐	☒	Eukaryotic cell lines
☒	☐	Palaeontology
☒	☐	Animals and other organisms
☐	☒	Human research participants
☒	☐	Clinical data

Methods

n/a	Involved in the study	
☒	☐	ChIP-seq
☒	☐	Flow cytometry
☒	☐	MRI-based neuroimaging

Antibodies

Antibodies used	For western blot analysis, primary antibodies against calretinin (Santa Cruz, SC-365956), PTCH1 (Abcam, ab53715), GLI1 (Abcam, ab134906), GLI2 (Origene, TA804601), Cyclin D1 (Cell Signaling, 92G2, #2978), FLAG M2 (Sigma, F1804), SUFU (Cell Signaling, #2522S) and tubulin (Cell Signaling, DM1A), and conjugated secondary anti-rabbit (LI-COR, 926-68073) and anti-mouse (LI-COR, 926-32212) antibodies were used. For immunofluorescence analysis, primary Calretinin (Santa Cruz, sc-565956) and anti-FLAG M2 (Sigma, F-1804) and secondary Alexa 647 goat anti-mouse (Life Technologies, A21236) antibodies were used.
Validation	The antibodies employed have been previously cited for the indicated use. Western blot/ immunofluorescence analyses were validated at the RNA level using quantitative RT-PCR.

Eukaryotic cell lines

Policy information about cell lines

Cell line source(s)	Immortalized mesenchymal stem cells (ASC52telo) from ATCC; HEK-293 cells from ATCC; DAOY medulloblastoma cells from ATCC; Human Basal Cell Carcinoma cells from CELPROGEN
Authentication	All cell lines employed in this study were authenticated using short tandem repeat profiling.
Mycoplasma contamination	All cell lines were routinely tested for mycoplasma using the PCR-based Universal Mycoplasma Detection kit (ATCC) and no mycoplasma was detected.

Commonly misidentified lines (See ICLAC register)	No commonly misidentified lines were used in this study.

Human research participants

Policy information about studies involving human research participants

Population characteristics	Archived tumors classified as of sclerosing stromal tumors of the ovary were selected for this study, and retrospectively analyzed.
Recruitment	Retrospective analysis of banked specimens. No prospective recruitment was performed.
Ethics oversight	The study was approved by the Institutional Review Boards (IRBs)/ local ethics committees of the authors' institutions, and patient consents were obtained following the respective IRB protocols approved by the authors' institutions.

Note that full information on the approval of the study protocol must also be provided in the manuscript.
